# Supplementary material for: Activation and In Vivo Evolution of the MAIT Cell Transcriptome in Mice and Humans Reveals Tissue Repair Functionality
Source: Cell Rep. 2019 Sep 17;28(12):3249–3262.e5. doi: 10.1016/j.celrep.2019.07.039 (PMC6859474; doi:10.1016/j.celrep.2019.07.039)
Supplement: Document S1. Figures S1–S7 [file mmc1.pdf]

Cell Reports, Volume *28*

Supplemental Information

Activation and *In Vivo* Evolution of the MAIT Cell Transcriptome in Mice and Humans Reveals Tissue Repair Functionality

Timothy S.C. Hinks, Emanuele Marchi, Maisha Jabeen, Moshe Olshansky, Ayako Kurioka, Troi J. Pediongco, Bronwyn S. Meehan, Lyudmila Kostenko, Stephen J. Turner, Alexandra J. Corbett, Zhenjun Chen, Paul Klenerman, and James McCluskey

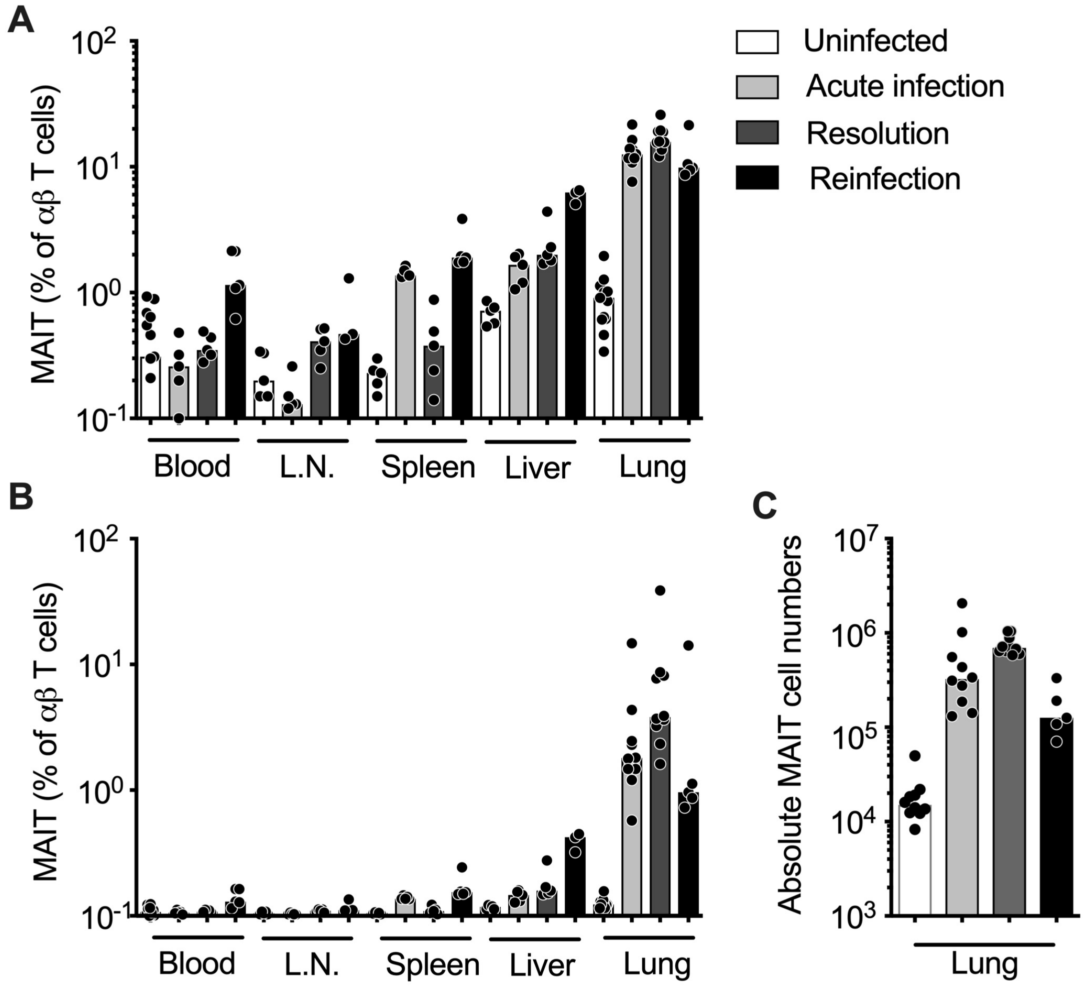

Supplementary figure S1. Tissue distribution of MAIT cells during infection *in vivo.* Related to Figure 1.

(**A, B**) Relative frequencies of MR1-5-OP-RU tetramer$^+$ MAIT cells as a proportion of total live TCRβ^+ T cells in the peripheral blood, mesenteric lymph node (L.N.), spleen, liver and lungs of C57BL/6 mice before, or after intranasal infection with 1x10^4 CFU *L. longbeachae*. Mice were sacrificed before ('uninfected'), or 7 days after infection ('acute'), or at least 12 weeks post infection ('resolution') or 7 days after a second intranasal infection with 2x10^4 CFU *L. longbeachae* in mice which had recovered from infection 12 weeks previously

(‘reinfection’). Graphs show combined data from experiments using 3-5 mice per group and performed one-three times. (**C**) Absolute numbers of pulmonary MAIT cells per mouse during the same experimental conditions, showing representative data from 5-10 mice per group performed twice. ANOVA $P<0.0001$, Tukey’s shows significant differences for all inter-group comparisons except acute v resolution.

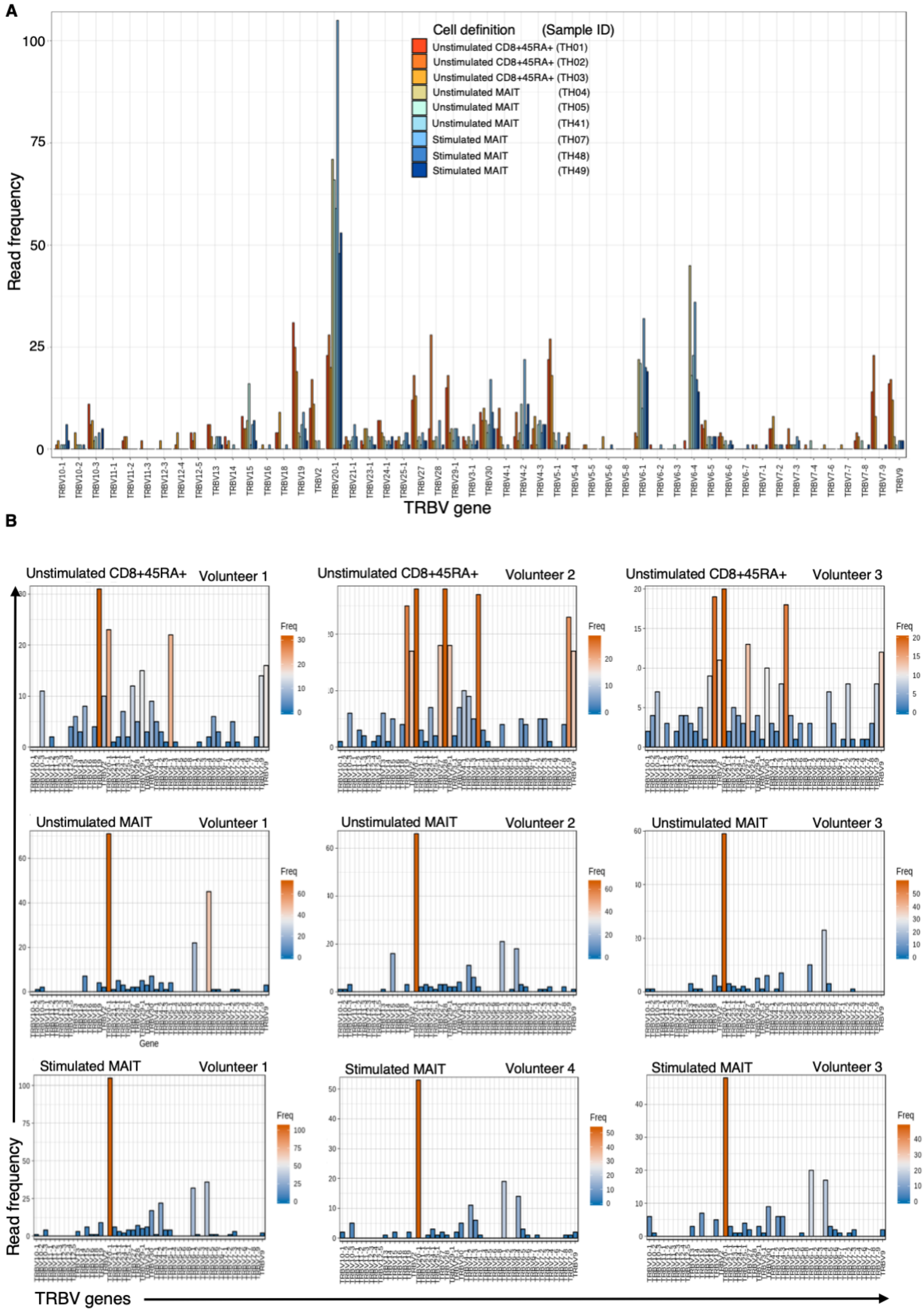

Supplementary figure S2. TCR repertoire analyses: human. Related to Table

2.

Analysis of TCR TRBV gene segment utilisation in human samples shown (**A**) aggregated and (**B**) for individual volunteers. RNA reads for TRBV genes from unstimulated human CD3+CD8+45RA+ T cells and MR1-5OP-RU-tetramer+ MAIT cells were compared for overall frequencies within the dataset. *y* axes show the absolute counts of gene segment usages in all clonetypes (unique CDR3 sequences identifying a unique cell clone) reconstructed. In MAIT cell populations increased read frequencies are observed for TRBV20-1, TRBV 6-1, and TRBV 6-4 in humans and for TRBV 19, TRBV 13-2 and TRBV 13-3 in mice, but with no specific repertoire changes observed between stages of infection. *n*=3 biological replicates / group performed once.

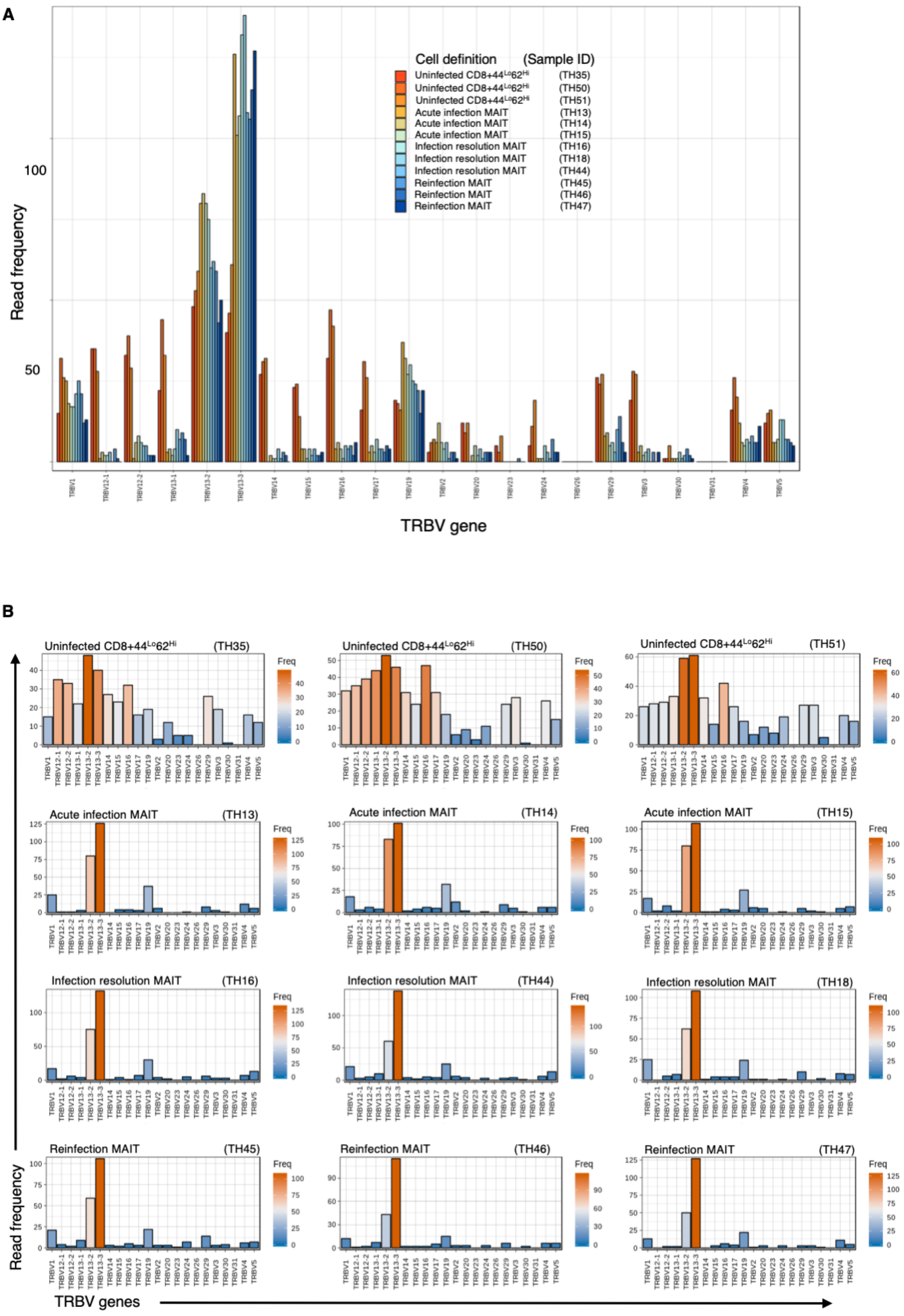

Supplementary figure S3. TCR repertoire: murine. Related to Table 2.

Analysis of TCR TRBV gene segment utilisation in murine samples shown (**A**) aggregated, where each naïve sample represents a pool from 3 animals, and (**B**) as individual replicates. RNA reads for TRBV genes from infection-naïve murine CD3+CD45.2+CD19-TCRβ+CD44-CD62L+ T cells and MR1-5OP-RU-tetramer+ MAIT cells were compared for overall frequencies within the dataset. *y* axes show the absolute counts of gene segment usages in all clonetypes (unique CDR3 sequences identifying a unique cell clone) reconstructed. In MAIT cell populations increased read frequencies are observed for TRBV 19, TRBV 13-2 and TRBV 13-3 in mice, but with no specific repertoire changes observed between stages of infection. *n*=3 biological replicates / group performed once.

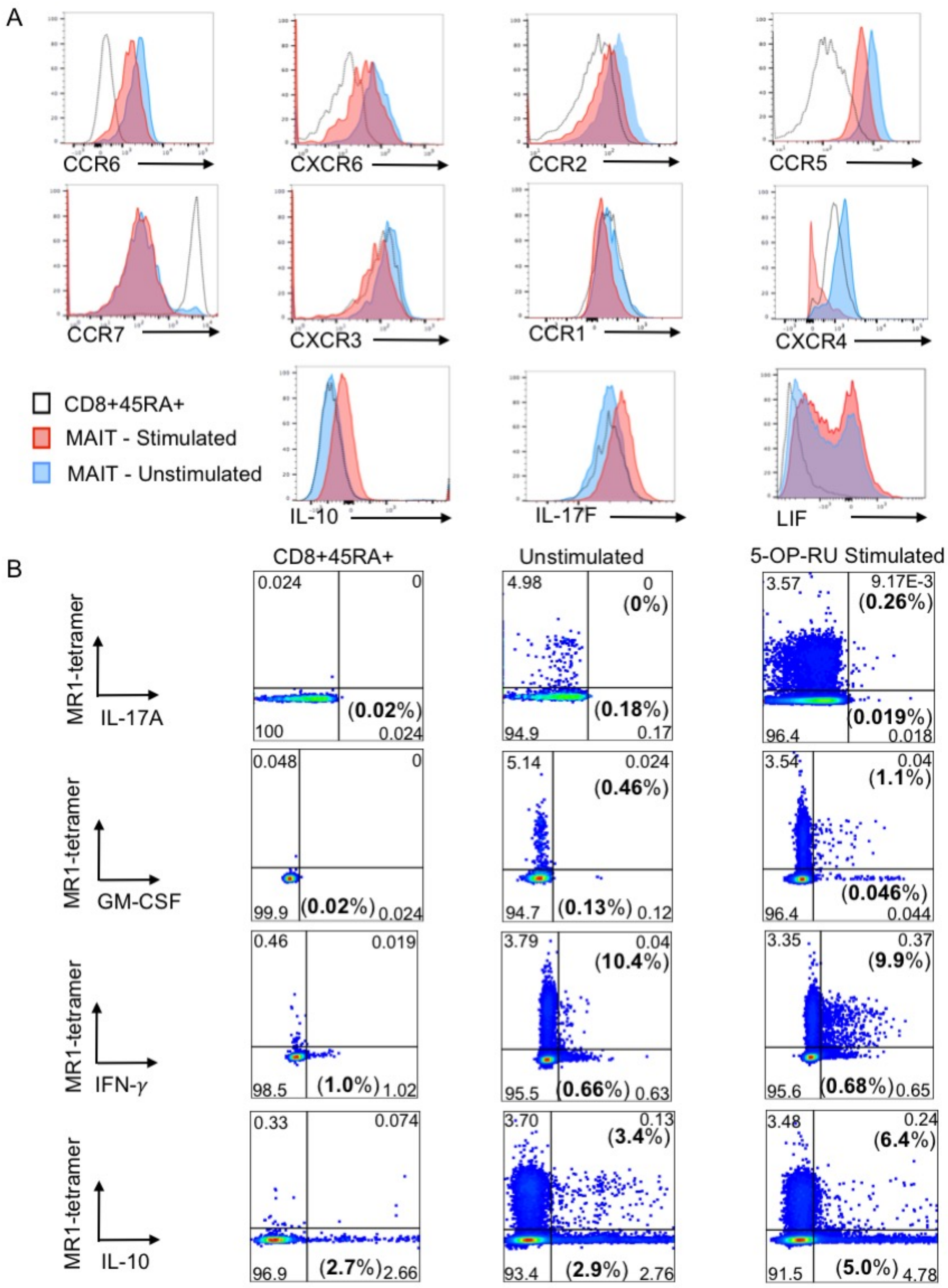

Supplementary figure S4. Cytometric validation of key differentially expressed genes (human). Related to Table 3.

(**A**) Representative flow-cytometry plots showing surface expression of the chemokine receptors CCR5, CCR7, CCR2, CCR1, CXCR6, CXCR4, CCR6, CXCR3, and intracellular

expression of the cytokines IL-10, IL-17F and LIF. Histograms compare staining of $CD8^+CD45RA^+$ cells (black, dotted) with unstimulated MAIT cells (blue) or MAIT cells after 6 h stimulation with 10 nM 5-OP-RU (red). (**B**) Representative flow-cytometry plots showing expression of the cytokines IL-17A, GM-CSF, IFN-γ and IL-10, by intracellular cytokine staining. Histograms compare staining of MR1-5-OP-RU $tetramer^+$ MAIT cells after 6 h stimulation with 10 nM 5-OP-RU in the presence of brefeldin A (right) with unstimulated cells (middle) and tetramer-negative, $CD8^+CD45RA^+$ naïve T cells (left). Figures in brackets represent percentage of MAIT cells (top / middle) or of $CD8^+CD45RA^+$ T cells (bottom) expressing the cytokine. Results are representative of three independent donors.

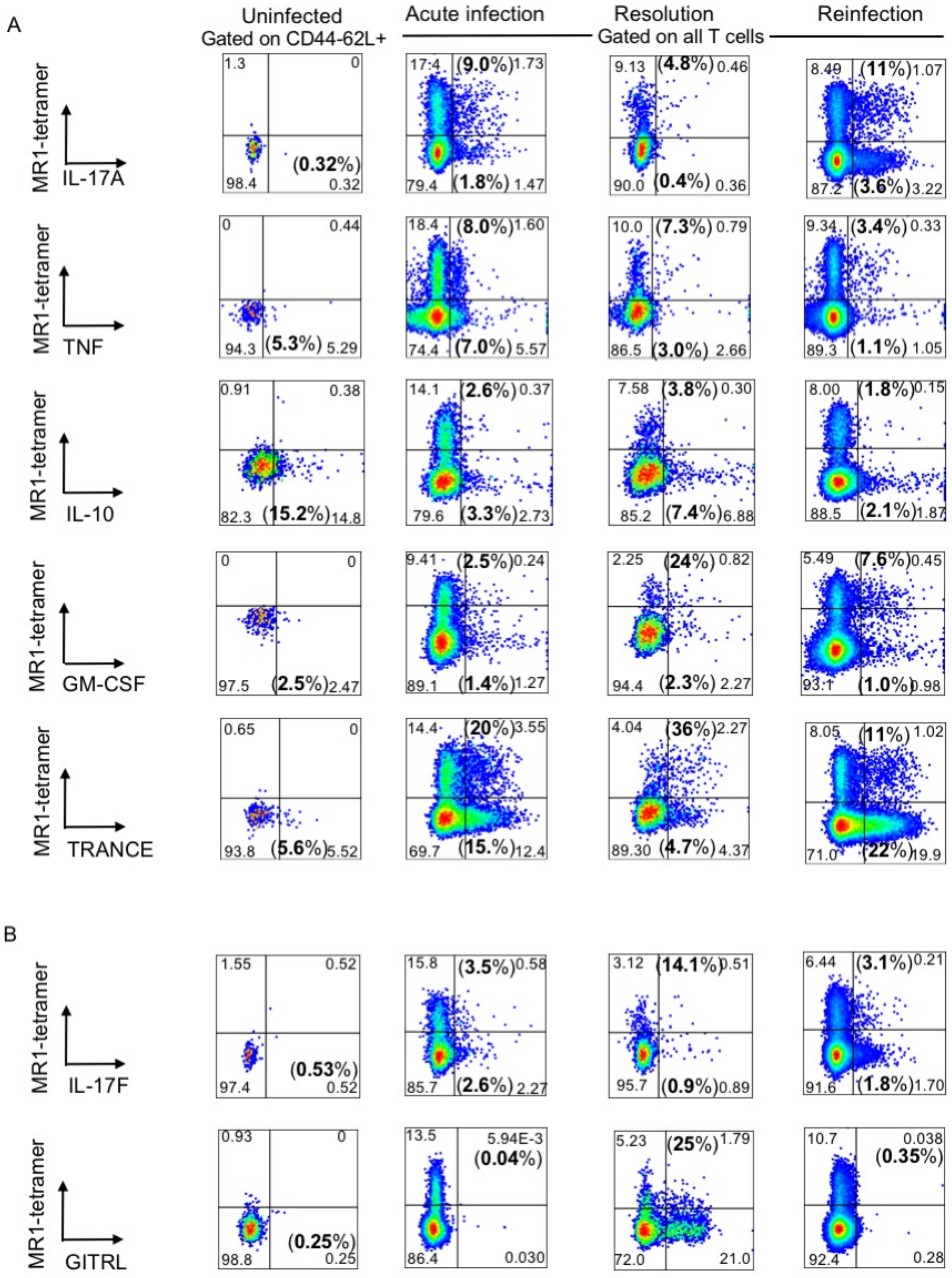

Supplementary figure S5. Cytometric validation of key differentially expressed genes (murine). Related to Table 2.

Representative flow-cytometry plots showing expression of the cytokines IL-17A, TNF, IL-10, TRANCE (TNFSF11), GM-CSF, IL-17F and GITRL (TNFSF18) by intracellular cytokine

staining on murine pulmonary T cells. Histograms compare staining of $CD44^{-}CD62L^{+}$ T cells from uninfected mice (left) with MR1-5-OP-RU tetramer^{+} MAIT cells either 7 days (‘acute’, middle left) or 12 weeks (‘resolution’, middle right) after infection with 1 x10^{4} CFU intranasal *L. longbeachae,* or 7 days after reinfection with 2 x10^{4} CFU i.n. *L. longbeachae* in mice previously infected 12 weeks prior with 10^{4} CFU i.n. *L. longbeachae* (‘reinfection’, right). Cells were incubated for 4 h in the presence of brefeldin A without (A, *ex vivo*) or with (B, stimulated) PMA and ionomycin. Figures in brackets represent percentage of $CD44^{-}CD62L^{+}$ T cells (left, lower quadrants) or MR1-tetramer^{+} MAIT cells (middle and right, upper quadrants), or MR1-tetramer^{-} conventional T cells (middle and right, lower quadrants) expressing the cytokine. Results are representative of three independent replicates performed on two separate days.

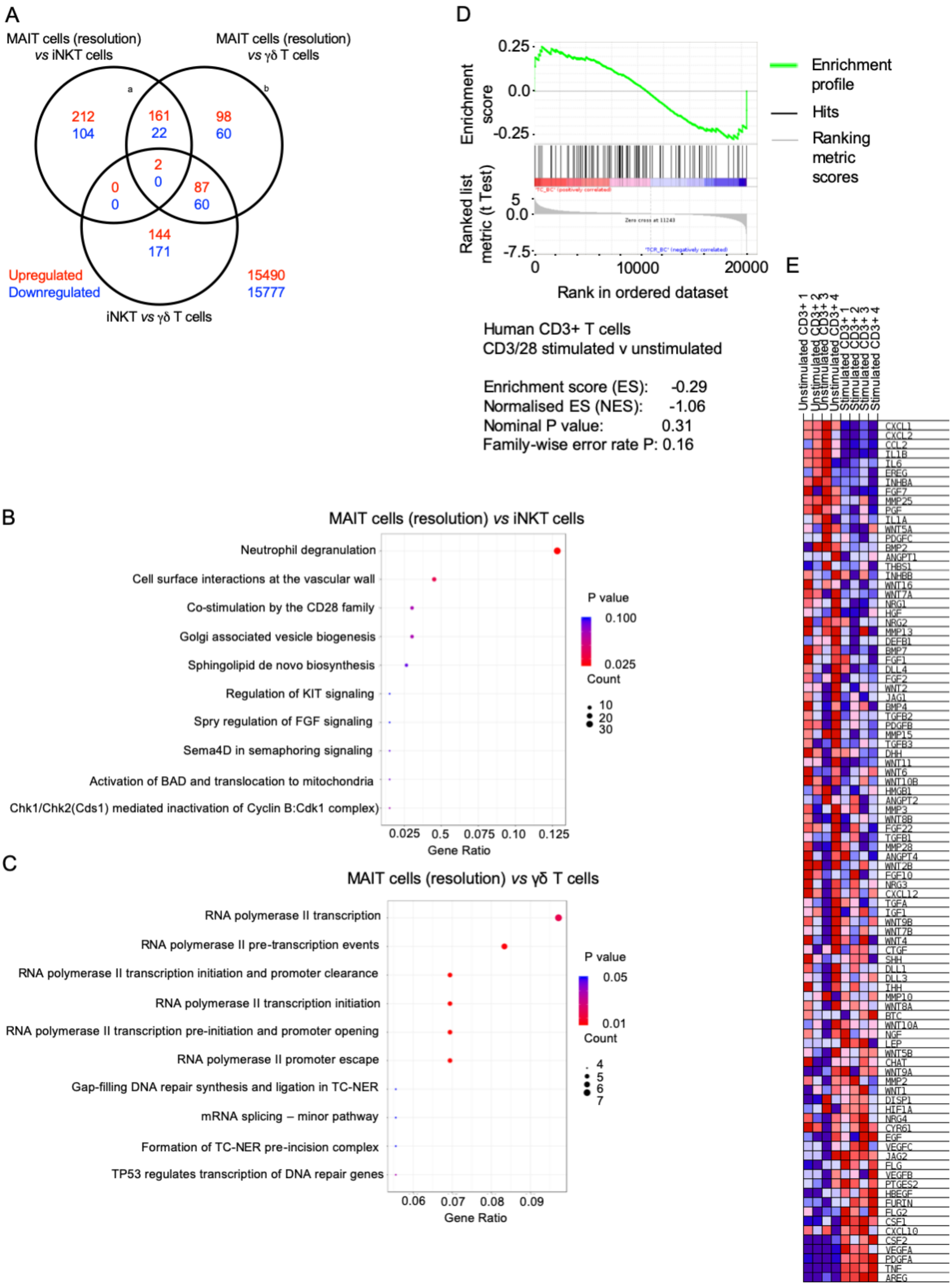

Supplementary figure S6. Additional analyses in murine MAIT, iNKT and γδ T cells, and human CD3+ cells. Related to Figure 2 and 4.

(A-C) Differential gene expression and Reactome pathway analysis comparing murine MAIT, iNKT and γδ T cells. (A) Venn diagram comparing numbers of differentially expressed genes between MAIT cells, iNKT cells and γδ T cells. Differential gene expression analysis was performed on transcriptomes of selected cell types shown in figure 3, comprising RNA seq data from this study and microarray data downloaded from the ImmGen database(Heng et al., 2008). MAIT cells comprised MR1-5-OP-RU tetramer+ MAIT cells at resolution of infection (12 weeks post infection). iNKT cells comprise all iNKT cell subsets shown in figure 3, excluding thymic precursor subsets: i.e. the ImmGen subsets: NKT.4-.Sp_1/2/3, NKT.4+/Sp1/2/3, NKT.4+.Lv_1/2/3/4, NKT.4-.Lv_1/2/3/4. Details of upregulated genes denoted (a) and (b) are provided in supplementary tables S15 and S16 respectively. (Red, upregulated; blue, downregulated). (**B**) Pathway analysis of the differential gene expression contrasts between murine lung MAIT cells at resolution of infection and non-thymic precursor iNKT cells shows that MAIT cells have upregulation of pathways associated with neutrophil degranulation and of cell surface interactions with vasculature. (**C**) Pathway analysis comparing murine lung MAIT cells at resolution of infection and γδ T cells. Upregulated MAIT cell pathways are those associated with RNA transcription. Plots show the extent to which named pathways from the curated Reactome database are upregulated. Colour intensity represents statistical significance of the upregulation, dot size represents the number of genes upregulated in the pathway, x axis represents the proportion of all differentially expressed genes included in the pathway (‘gene ratio’). *n*=3 biological replicates / group performed once.

(**D-E**) Gene set enrichment analysis for tissue repair gene signature in human CD3+ T cells Gene set enrichment analysis (GSEA) was used to investigate potential enrichment of the same tissue repair signature presented in Figure 4(Linehan et al., 2018) in publicly available gene expression profiles from human CD3+ T cells (GEO Series accession no. GSE13887)(Fernandez et al., 2009). CD3+ T cells were negatively isolated with a Dynal kit

and, in the case of stimulated cells, Stimulated through CD3/CD28 for 24hr and gene expression analysed using Affymetrix Human Genome U133 Plus 2.0 Array. GSEA was performed on 4 replicates each of stimulated and unstimulated samples. (**A**) GSEA summary plots. The gene set is not significantly enriched: enrichment score (ES) = -0.28, normalised enrichment score (NES) = -1.06, family-wise error rate (FWER) p=0.16, nominal p value =0.3. (**B**) Heat map of expression of tissue repair genes (red, highest expression, blue, lowest). *n*=3 biological replicates / group performed once.

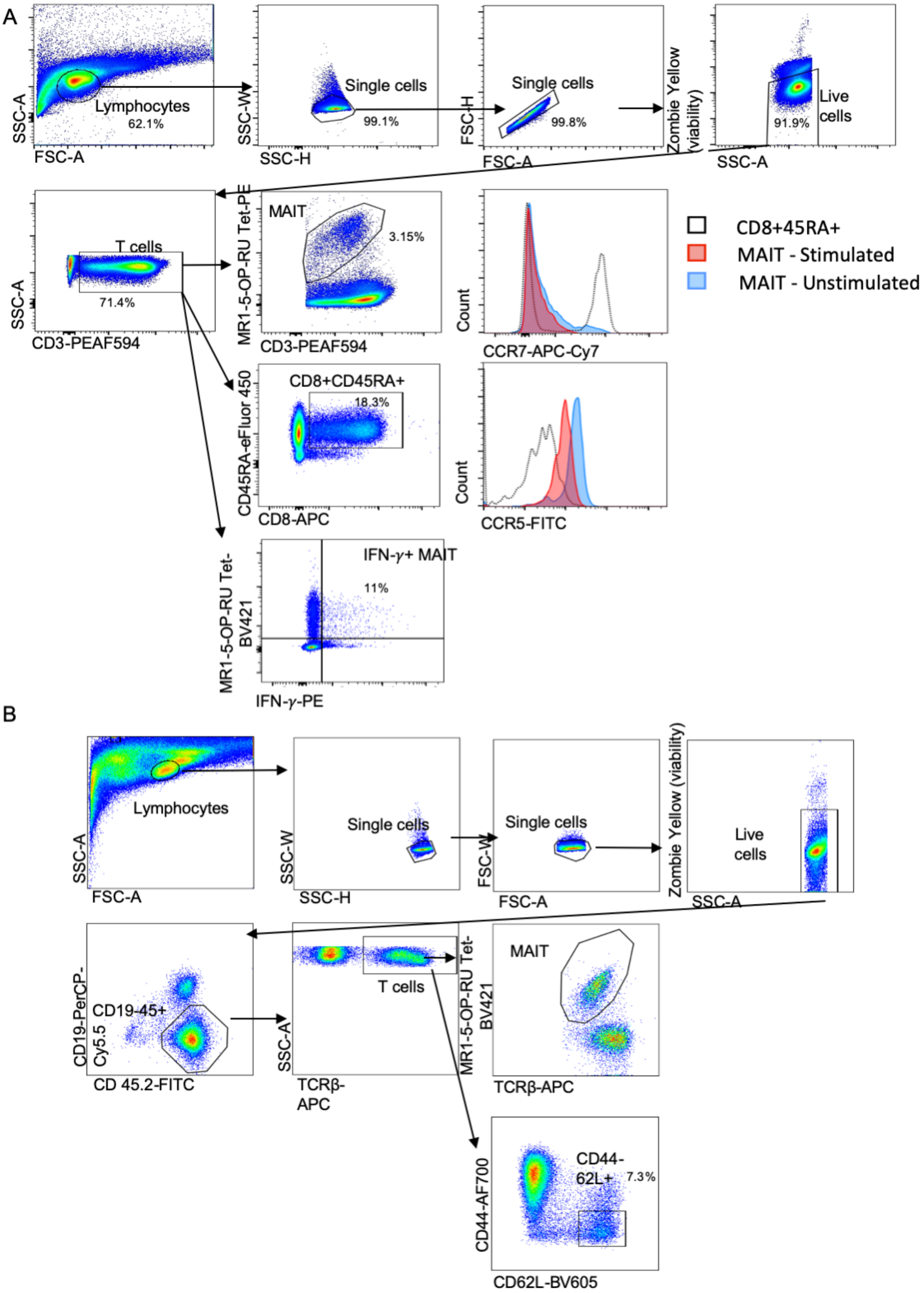

Supplementary figure S7. Cytometric gating strategies. Related to Table 1.

(**A**) Cytometric gating strategy (human). Human peripheral blood lymphocytes were identified, and doublets excluded, using forward and side scatter characteristics. Dead cells were excluded

using Zombie Yellow viability stain, then populations were gated on $CD3^+$ cells, then on either MR1-5-OP-RU tetramer conjugated to PE or BV421 for MAIT cells, or on CD8 and CD45RA to identify naïve $CD8^+CD45RA^+$ cells. Histograms compare staining of $CD8^+CD45RA^+$ cells (black) with unstimulated MAIT cells (blue) or MAIT cells after 6 h stimulation with 10 nM 5-OP-RU (red). For intracellular cytokines, where basal cytokine secretion was minimal, gates were set on the unstimulated MAIT cell sample.

(**B**) Cytometric gating strategy (murine). Related to Table 2. Pulmonary lymphocytes were identified, and doublets excluded, using forward and side scatter characteristics. Dead cells were excluded using Zombie Yellow viability stain, then populations were gated on $CD19^-$ $CD45.2^+$ cells, then TCRβ+ cells and finally MR1-5-OP-RU tetramer$^+$ MAIT cells, or $CD44^-$ $CD62L^+$ T cells.
