# Supplementary material for: Activation and In Vivo Evolution of the MAIT Cell Transcriptome in Mice and Humans Reveals Tissue Repair Functionality
Source: Cell Rep. 2019 Sep 17;28(12):3249–3262.e5. doi: 10.1016/j.celrep.2019.07.039 (PMC6859474; doi:10.1016/j.celrep.2019.07.039)
Supplement: Document S2. Article plus Supplemental Information [file mmc12.pdf]

Cell Reports

Resource

Activation and *In Vivo* Evolution of the MAIT Cell Transcriptome in Mice and Humans Reveals Tissue Repair Functionality

Graphical Abstract

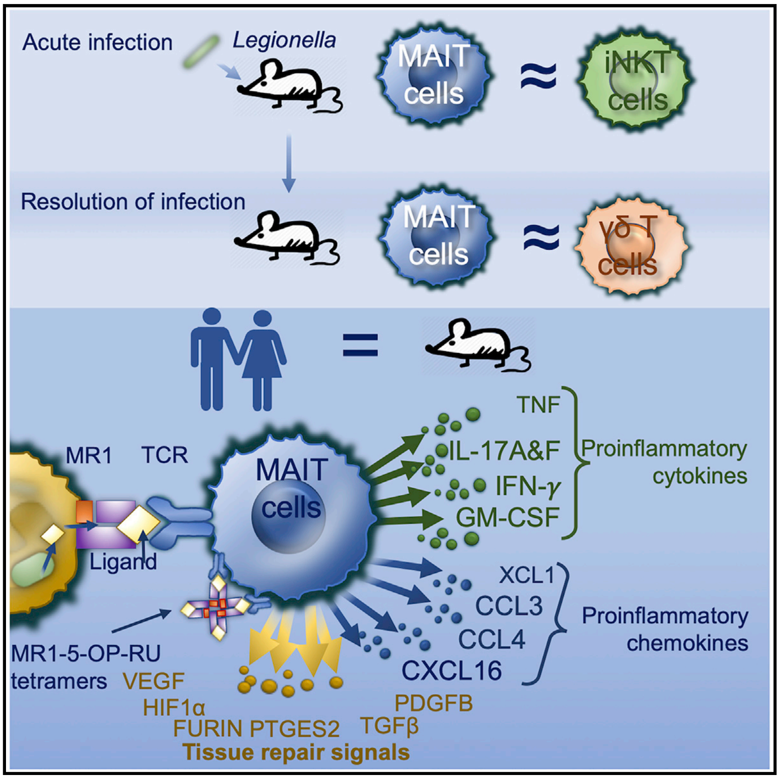

Highlights

- We define the basic transcriptome of an activated MAIT cell in mice and humans
- During acute infection, the MAIT cell transcriptome is most similar to iNKT cells
- After the resolution of infection, MAIT cells more closely resemble γδ T cells
- Both human- and murine-activated MAIT cells express a strong tissue repair signature

Authors

Timothy S.C. Hinks, Emanuele Marchi, Maisha Jabeen, ..., Zhenjun Chen, Paul Klenerman, James McCluskey

Correspondence

timothy.hinks@ndm.ox.ac.uk

In Brief

Mucosal-associated invariant T (MAIT) cells are implicated in antibacterial and antiviral immunity. Using RNA sequencing of human MAIT cells stimulated with their cognate ligand and murine MAIT cells stimulated by acute *Legionella* infection, Hinks et al. report that activation leads to expression of a strong tissue repair signature in both species.

Hinks et al., 2019, Cell Reports *28*, 3249–3262
September 17, 2019 © 2019 The Author(s).
https://doi.org/10.1016/j.celrep.2019.07.039

CellPress

Cell Reports
Resource

OPEN ACCESS
CellPress

Activation and *In Vivo* Evolution of the MAIT Cell Transcriptome in Mice and Humans Reveals Tissue Repair Functionality

Timothy S.C. Hinks,[1,2,6,*] Emanuele Marchi,[3] Maisha Jabeen,[2] Moshe Olshansky,[1,4] Ayako Kurioka,[3] Troi J. Pediongco,[1] Bronwyn S. Meehan,[1] Lyudmila Kostenko,[1] Stephen J. Turner,[4] Alexandra J. Corbett,[1] Zhenjun Chen,[1] Paul Klenerman,[3,5] and James McCluskey[1]
[1]Department of Microbiology and Immunology, Peter Doherty Institute for Infection and Immunity, University of Melbourne, Melbourne, VIC 3000, Australia
[2]Respiratory Medicine Unit, Nuffield Department of Medicine, University of Oxford, OX3 9DU, Oxfordshire, UK
[3]Peter Medawar Building for Pathogen Research and Translational Gastroenterology Unit, Nuffield Department of Clinical Medicine, University of Oxford, OX1 3SY, Oxfordshire, UK
[4]Infection and Immunity Program and The Department of Biochemistry and Molecular Biology, Biomedicine Discovery Institute, Monash University, Clayton, VIC 3800, Australia
[5]Translational Gastroenterology Unit, Level 5 John Radcliffe Hospital, OX3 9DU, Oxfordshire, UK
[6]Lead Contact
*Correspondence: timothy.hinks@ndm.ox.ac.uk
https://doi.org/10.1016/j.celrep.2019.07.039

SUMMARY

Mucosal-associated invariant T (MAIT) cells are MR1-restricted innate-like T cells conserved across mammalian species, including mice and humans. By sequencing RNA from sorted MR1-5-OP-RU tetramer$^+$ cells derived from either human blood or murine lungs, we define the basic transcriptome of an activated MAIT cell in both species and demonstrate how this profile changes during the resolution of infection and during reinfection. We observe strong similarities between MAIT cells in humans and mice. In both species, activation leads to strong expression of pro-inflammatory cytokines and chemokines as well as a strong tissue repair signature, recently described in murine commensal-specific H2-M3-restricted T cells. Transcriptomes of MAIT cells and H2-M3-specific CD8+ T cells displayed the most similarities to invariant natural killer T (iNKT) cells when activated, but to γδ T cells after the resolution of infection. These data define the requirements for and consequences of MAIT cell activation, revealing a tissue repair phenotype expressed upon MAIT cell activation in both species.

INTRODUCTION

Mucosal-associated invariant T (MAIT) cells are innate-like T cells that express a "semi-invariant" αβ T cell receptor (TCR) and recognize metabolic derivatives of riboflavin biosynthesis (Corbett et al., 2014; Eckle et al., 2015; Kjer-Nielsen et al., 2012) presented on the restriction molecule major histocompatibility complex (MHC)-related protein-1 (MR1) (Porcelli et al., 1993; Tilloy et al., 1999). These antigens, which include the potent MAIT cell ligand 5-(2-oxopropylideneamino)-6-D-ribitylaminouracil (5-OP-RU) (Corbett et al., 2014; Mak et al., 2017), are produced by a wide variety of bacteria, mycobacteria, and yeasts (Chua et al., 2012; Gold et al., 2013; Kjer-Nielsen et al., 2012; Le Bourhis et al., 2010; Maggio et al., 2015) but are absent from mammals and therefore allow host-pathogen discrimination. MAIT cells have a strong pro-inflammatory phenotype and produce interferon-γ (IFN-γ), tissue necrosis factor (TNF), and IL-17A after phorbol myristate acetate (PMA) and ionomycin stimulation (Dusseaux et al., 2011) or after infection (Wang et al., 2018).

While baseline frequencies of MAIT cells are low in specific-pathogen-free C57BL/6 mice, we, among others, have previously shown that MAIT cells can be activated and expand *in vivo* in response to a pulmonary infection with specific intracellular bacteria expressing the riboflavin pathway—*Salmonella* Typhimurium (Chen et al., 2017), *Legionella spp* (Wang et al., 2018), and *Francisella tularensis* (Meierovics et al., 2013; Meierovics and Cowley, 2016)—or in response to synthetic 5-OP-RU accompanied by a Toll-like receptor agonist (Chen et al., 2017), providing valuable models to dissect MAIT cell biology.

To date, the requirements for TCR-dependent activation of MAIT cells *in vivo* have not been systematically characterized, nor have the consequences of such activation been fully defined. Here we have used MR1 tetramers (Corbett et al., 2014) loaded with 5-OP-RU to specifically identify MAIT cells from human peripheral blood and murine lungs, allowing us to assess the requirements for, and consequences of, MAIT cell activation *ex vivo* and *in vivo*. Using a transcriptomic approach on sorted MR1-5-OP-RU tetramer$^+$ cells, we define the transcriptome of an activated MAIT cell in both species and explore how this changes during the resolution of infection and during reinfection.

Our data reveal strong similarities between MAIT cells in humans and in mice at a transcriptional level; show that MAIT cells displayed the closest similarities to invariant natural killer T (iNKT) cells when activated, but after the resolution of infection were

This is an open access article under the CC BY license (http://creativecommons.org/licenses/by/4.0/).

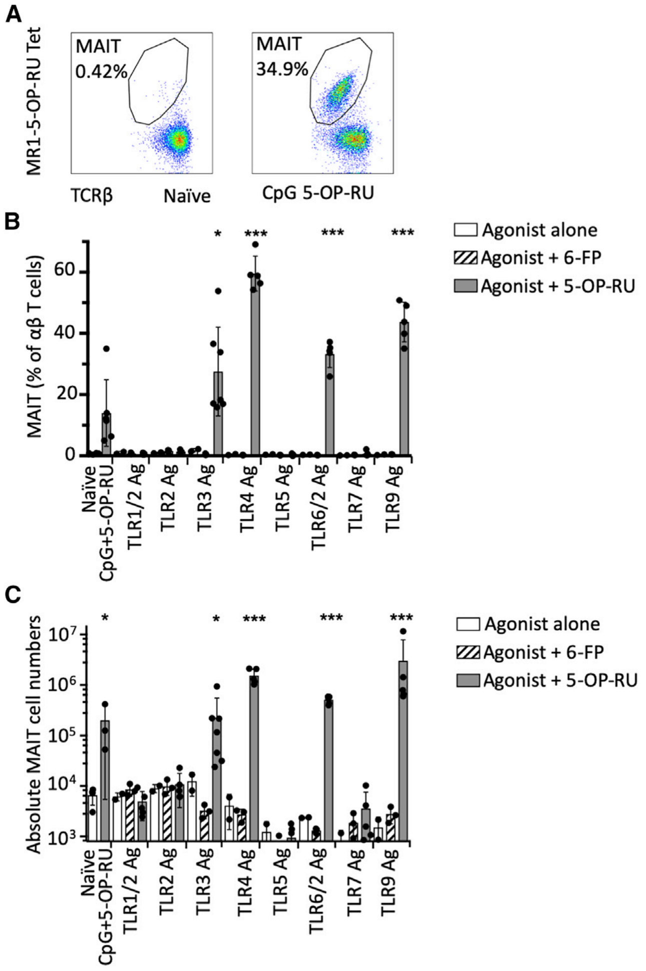

Figure 1. Costimulatory Requirements for MAIT Cell Activation *In Vivo*

(A) Representative flow-cytometry plots showing MAIT cell percentage among TCRβ$^+$ lymphocytes in the lungs of C57BL/6 mice with or without prior stimulation with intranasal CpG and 5-OP-RU.

(B and C) Relative (B) and absolute (C) numbers of MR1-5-OP-RU tetramer$^+$ MAIT cells in the lungs of C57BL/6 mice 7 days after intranasal exposure to specific TLR agonists either alone or in combination with 76 pmol 6-FP, or with 76 pmol 5-OP-RU. Control mice received nothing (n = 4, naive) or CpG with 5-OP-RU (n = 3). Experiments used n = 5 (5-OP-RU treated), n = 3 (6-FP treated), or n = 2 (TLR agonist alone) mice per group. The experiment was subsequently repeated with similar results. Statistical tests: unpaired t tests, comparing TLR + 5-OP-RU with naive control (n = 4), on untransformed (B) or log-transformed (C) data with Bonferroni corrections *$p < 0.05$, ***$p < 0.001$.

more comparable to γδ T cells; and reveal a previously unknown tissue repair phenotype expressed upon MAIT cell activation in both species.

RESULTS

Activation Requirements of MAIT Cells *In Vivo*

First, we aimed to test, systematically, the activation requirements of MAIT cells *in vivo* in mouse lungs. We have previously shown that pulmonary MAIT cell frequencies in mice can be markedly enhanced by intranasal administration of 5-OP-RU if it is co-administered with S-[2,3-bis(palmitoyloxy)propyl] cysteine (Pam2Cys), CpG ODN 1668, or polyinosinic:polycytidylic acid (poly I:C), which are agonists for TLR2/6, TLR9, and TLR3, respectively. We therefore investigated agonists for each of the murine TLRs, using the maximum doses presented in a literature review of previous studies of these compounds. All animals received the relevant TLR intranasally on day 0. In experimental animals, this was administered in combination with 76 pmol 5-OP-RU on day 0, with repeated inoculae of 76 pmol 5-OP-RU on days 1, 2, and 4. Control mice received the same TLR ligand and 76 pmol of the non-activating MR1 ligand 6-formyl pterin (6-FP) (Kjer-Nielsen et al., 2012) according to the same schedule, or the TLR ligand alone (Table S1). We observed 15- to 180-fold enrichment of pulmonary MAIT cell frequencies (defined as CD3$^+$CD45.2$^+$CD19$^-$MR1-5-OP-RU tetramer$^+$ cells) at day 7, after administration of 5-OP-RU with agonists of TLR3 (high molecular weight poly I:C), TLR4 (lipopolysaccharide from *E.coli*), TLR2/6 (FSL-1 [Pam2CGDPKHPKSF]), and TLR9 (CpG ODN1826), but not with agonists of TLR1/2 (Pam3CSK4), TLR2 (heat killed *Listeria monocytogenes*), TLR5 (flagellin from *S.* Typhimurium), or TLR7 (Imiquimod) (Figure 1), suggesting there is a specific and restricted range of danger signals that are capable of providing the necessary co-stimulus to drive MAIT cell accumulation in response to 5-OP-RU antigens.

Transcriptomic Profile of Activated Human and Murine MAIT Cells

Having defined the minimum requirements for TCR-mediated activation of MAIT cells, we sought to describe in detail the consequences of their activation using a transcriptomic approach to define the basic transcriptome of a MAIT cell in both humans and mice and to determine how this is modulated by activation. Fresh human peripheral blood cells were obtained from three donors. These were cultured for 6 h with (“stimulated”) or without (“unstimulated”) 10 nM 5-OP-RU; magnetically enriched on MR1-tetramer$^+$ cells; and flow-sorted for RNA sequencing of live CD3$^+$TCR Vα7.2$^+$ MR1-5-OP-RU tetramer+ MAIT cells, and of unstimulated naive live CD8$^+$CD45RA$^+$ T cells as a comparator cell type (Table S2). This cell type was selected as a comparator to enhance the contrast with MAIT cells and allow a more comprehensive characterization of the MAIT cell’s full functional phenotype.

We have previously shown that pulmonary infection of mice with the intracellular pathogen *Legionella longbeachae* induces strong TCR-mediated MAIT cell activation, and this plays a significant role in host immune protection (Wang et al., 2018), thus constituting a physiologically relevant model of *in vivo* MAIT cell activation. This model infection induces a rapid and sustained expansion of MAIT cells in the lungs (Figure S1), which closely parallels the MAIT cell expansion observed with TCRs and specific TLR ligation in Figure 1. Using this model, we therefore included within the same sequencing experiment live pulmonary CD3$^+$45.2$^+$19$^-$MR1-5-OP-RU tetramer$^+$ MAIT cells, which were magnetically enriched and flow-sorted from the lungs of mice 7 days after infection with 1 × 10^4 CFU *L. longbeachae* (“acute”), or at least 12 weeks post infection (“resolution”) or 7 days after a second intranasal infection with 2 × 10^4 CFU

L. longbeachae in mice that had recovered from infection 12 weeks previously ("reinfection"). Because of the very low numbers of MAIT cells in uninfected, specific-pathogen-free mice (Rahimpour et al., 2015), it was not possible to obtain robust data on naive MAIT cells from such animals. Instead, live $CD3^{+}CD45.2^{+}CD19^{-}CD8^{+}CD44^{-}CD62L^{+}$ naive T cells from uninfected mice were used as a comparator cell type, with the objective of providing a comprehensive characterization of MAIT cells' phenotypic and functional capabilities.

The number of differentially expressed genes (DEGs) in activated MAIT cells, compared with naive $CD8^{+}$ T cells, was 4,613 genes in human 5-OP-RU-stimulated MAIT cells and 3,758 genes in acutely infected mice at a false discovery rate (FDR) p value of < 0.05 and minimum $\log_{(2)}$ fold change of ± 1. (Numbers and full lists of DEGs are shown in Table S3.) These genes constitute the basic transcriptome of an activated MAIT cell in each species. To explore the nature of these gene profiles further, we compared different activation states of MAIT cells. In humans, 3,227 genes were differentially expressed between stimulated and unstimulated MAIT cells and could therefore be considered the direct signature of TCR-mediated MAIT cell activation, while 968 genes were differentially expressed between unstimulated MAIT cells and naive $CD8^{+}$ T cells and therefore are more related to constitutive differences between T cell lineages (Table S3). In mice, 1,889 genes were differentially expressed between acute infection and the resolution of infection, analogous to the signature of TCR-mediated activation (Table S3).

Analysis of TCR genes over-represented in MAIT cells confirmed highly significant, selective use of TRAV1-2 with T cell receptor beta variable (TRBV)6-4, TRBV6-1, and TRBV20-1 in humans and Trbv13-3 with Trav1 and Traj33 in mice, in MAIT cells compared to naive CD8 T cells, as expected (Lepore et al., 2014; Porcelli et al., 1993; Reantragoon et al., 2013; Tilloy et al., 1999) (Table S4; Figures S2 and S3). We did not observe a specific change in the MAIT cell TRBV repertoire between stages of infection.

Focused analysis of known cytokine genes confirmed a strong upregulation of several pro-inflammatory type 1 and type 17 cytokines, especially CSF2 (GM-CSF), IL-17A, LIF, TNF, IFN-γ, and IL-17F, which were highly upregulated in both mice and humans in activated MAIT cells (Tables 1 and 2). Expression of selected cytokines was confirmed by flow cytometry (Figures S4 and S5). Likewise, there was significant, but more modest, upregulation of LTA (lymphotoxin A) and CSF1 (M-CSF) in both species. Some features were observed only in one species, notably IL-2 and TNSF14 (LIGHT) produced by activated human MAIT cells and Tnfsf11 (TNF-related activation-induced cytokine E [TRANCE], receptor activator of nuclear factor kappa-B ligand [RANKL]) by murine MAIT cells. In contrast to activation-induced cytokines, expression of the anti-apoptotic cytokine IL-15, implicated in the development and maturation of memory $CD8^{+}$ T cells (Jabri and Abadie, 2015), was restricted to MAIT cells in their resting states: human unstimulated MAIT cells or murine MAIT cells at the resolution of infection. In mice, the resolution of infection was also associated specifically with a strong expression of Tnfsf18 (glucocorticoid-induced TNF-related ligand [GITRL]), confirmed by flow cytometry. Expression of cytokine receptors is shown in Table S5, and that of recognized "CD markers" is shown in Table S6.

Similar analysis of chemokines showed strong activation-induced upregulation of a range of chemokines, including XCL1, CCL3 (MIP1α), CCL4 (MIP1β), and CXCL16, common to both species (Table S7), and of a common array of chemokine receptors CCR6, CXCR6, CCR1, CCR2, and CCR5 (Table S8; Figure S4), underlining a marked evolutionary conservation of MAIT cell function.

Pathway Analysis of the MAIT Cell Transcriptome

To analyze the transcriptome at the level of pathways, rather than individual genes, we looked for upregulation of pathways using the open-source, manually curated, peer-reviewed Reactome database (Fabregat et al., 2018). The main pathways upregulated in human 5-OP-RU stimulated MAIT cells, compared with naive $CD8^{+}CD45RA^{+}$ T cells, were related to endoplasmic reticulum stress—the unfolded protein response—and the related pathways IRE-1-α activation of chaperones and XBP1(S) activation of chaperones to chemokine receptor-ligation and to cholesterol biosynthesis (Figure 2A). When stimulated, human MAIT cells were contrasted directly with unstimulated MAIT cells: the activation of chemokine and cytokine signaling pathways—chemokine receptor-ligation, IL-2 signaling, and interleukin receptor Src homology and collagen (SHC) signaling—was more apparent, as was human solute carrier-mediated transmembrane transport (Figure 2B).

Perhaps reflective of the different context of activation, and consistent with the rapid MAIT cell expansion observed with infection *in vivo* (Meierovics et al., 2013; Wang et al., 2018), the murine MAIT cells activated by the acute *L. longbeachae* infection showed a very strong activation of cell cycle pathways, as well as signaling by RHO guanosine triphosphatases (GTPases) and chemokine receptor-ligation, with similar dominance of the cell cycle when MAIT cells activated by acute infection were contrasted directly with unstimulated MAIT cells after infection resolution (Figures 2C and 2D).

Comparison of MAIT Cell Transcriptomic Profile with Other T Cell Subsets

MAIT cells are a relatively ancient T cell subset (Yamaguchi et al., 1997), with both innate and adaptive properties, and they are capable of expressing diverse functions depending on the nature of the pathogenic encounter (van Wilgenburg et al., 2018; Wang et al., 2018). Therefore, we sought next to explore the nature of the murine MAIT cell transcriptome by comparing it with the transcriptional profiles for a wide range of other cell types reported within the Immunological Genome Project database (Heng et al., 2008) (Table S2). Using hierarchical clustering, while the pulmonary naive $CD8^{+}CD44^{-}CD62^{+}$ T cells clustered with the reference naive $CD8^{+}$ splenic T cells, activated MAIT cells from acute primary infection or from acute reinfection clustered most closely to iNKT cells (Figure 3). By contrast, after resolution of the infection, MAIT cells clustered most closely with unstimulated splenic $\gamma\delta$ T cells. We explored what transcriptional processes might drive this difference through analysis of differentially expressed genes (Table S9) and by using Reactome pathway analysis (Figure S6A). MAIT cells differed from non-thymic precursor

Table 1. Differentially Expressed Cytokine Genes: Human

Stimulated MAIT versus Unstimulated CD8+45RA+						Stimulated MAIT versus Unstimulated MAIT						Unstimulated MAIT versus Unstimulated CD8+45RA+					
Gene	Log Fold Change	Log CPM	LR	p Value	FDR p Value	Gene	Log Fold Change	Log CPM	LR	p Value	FDR p Value	Gene	Log Fold Change	Log CPM	LR	p Value	FDR p Value
CSF2*	15	7.4	62	3E−15	0.00	CSF2	15	7.4	64	1E−15	0.00	LIF	4.7	1.8	7.2	0.007	0.02
IL17A*	12	4.9	74	8E−18	0.00	IL17A	12	4.9	77	1E−18	0.00	TGFA	4.4	3.3	43	6E−11	0.00
IL2	11	4.0	42	1E−10	0.00	IFNG	9.7	8.4	61	6E−15	0.00	TNF	2.4	9.0	7.9	0.005	0.02
LIF*	8.9	1.8	26	3E−07	0.00	IL17F	8.3	1.2	35	3E−09	0.00	TNFSF13B	2.3	3.3	9.5	0.002	0.01
TNF*	8.7	9.0	59	2E−14	0.00	IL2	8.0	4.0	33	1E−08	0.00	TNFSF14	2.1	8.0	22	2E−06	0.00
IFNG*	8.4	8.4	51	1E−12	0.00	TNF	6.3	9.0	38	8E−10	0.00	CSF1	2.0	5.1	21	7E−06	0.00
IL17F*	8.3	1.2	32	1E−08	0.00	LIF	4.2	1.8	12	0.0005	0.00	IL15	1.9	2.3	5.8	0.02	0.04
TNFSF14	5.3	8.0	113	2E−26	0.00	IL10	4.1	1.5	14	0.0002	0.00	IL24	−1.8	2.4	6.2	0.01	0.04
CSF1*	5.2	5.1	120	7E−28	0.00	TNFSF14	3.2	8.0	52	6E−13	0.00	CXCR2	−4.0	3.0	9.7	0.001	0.01
IL26	4.6	1.8	16	7E−05	0.00	CSF1	3.2	5.1	59	2E−14	0.00	CXCR1	−4.1	2.6	14	0.0001	0.00
LTA*	3.3	7.0	48	4E−12	0.00	LTA	3.1	7.0	43	7E−11	0.00						
TGFA	2.9	3.3	19	1E−05	0.00	IL16	−1.1	9.3	5.6	0.018	0.04						
TNFSF13B	2.6	3.2	12	0.0004	0.00	TGFA	−1.6	3.3	7.4	0.006	0.02						
IL23A	2.3	4.7	12	0.0004	0.00	IL32	−1.6	9.7	20	8.1E−06	0.00						
IL32	−1.2	9.7	12	0.0005	0.00	IL18BP	−3.0	5.1	40	2.9E−10	0.00						
IL16	−1.5	9.3	11	0.001	0.00	CXCR1	−5.7	2.6	13	0.0004	0.00						
IL24	−2.0	2.4	7.7	0.005	0.01												
IL18BP	−3.6	5.1	57	6E−14	0.00												
CXCR2	−4.9	3.0	13	0.0002	0.00												
CXCR1	−9.8	2.6	37	1E−09	0.00												

Genes shown are censored at FDR $p \leq 0.05$ and log(2) fold change of ±1 and ordered by log fold change. CPM, counts per million; FDR, false discovery rate; LR, likelihood ratio.
*Genes are differentially expressed in both humans and mice.

Table 2. Differentially Expressed Cytokine Genes: Mouse

Acute Infection MAIT Cells versus Uninfected $CD8^{+}44^{Lo}62^{Hi}$						Resolved Infection MAIT Cells versus Acute Infection MAIT cells						Reinfection MAIT Cells versus Acute Infection MAIT Cells					
Gene	Log Fold Change	Log CPM	LR	p value	FDR p Value	Gene	Log Fold Change	Log CPM	LR	p Value	FDR p Value	Gene	Log Fold Change	Log CPM	LR	p Value	FDR p Value
Il17a*	14	7.3	316	9E−71	1E−68	Tnfsf18 (GITRL)	5.9	−0.52	14	0.0002	0.001	Csf1 (M-CSF)	2.2	3.0	12	0.0006	0.01
Ifng*	12	5.9	222	3E−50	2E−48	Tnfsf11 (TRANCE)	3.2	6.6	67	3E−16	4E−14	Il6st	1.5	6.7	14	0.0002	0.005
Csf2*	11	4.8	208	4E−47	3E−45	Il15	1.9	0.88	6.7	0.0096	0.03	Tnsf11 (TRANCE)	1.3	6.6	14	0.0002	0.007
Il17f*	11	4.9	171	5E−39	2E−37	Tgfb3	1.4	4.0	16	6E−05	0.0005	Il17a	–1.5	7.3	15	9.7E–05	0.003
Lif*	10	3.6	132	1E−30	5E−29	Il6st	1.3	6.7	9.9	0.002	0.008	Csf4	–1.2	4.8	12	0.0006	0.01
Il22	9.1	2.4	43	4E−11	4E−10	Il17f	1.1	4.9	8.5	0.004	0.02						
Il21	7.5	0.65	23	2E−06	1E−05	Il21	−3.8	0.65	11	0.0008	0.005						
Tnf*	4.5	6.0	188	1E−42	6E−41	Il17a	−2.4	7.3	38	9E−10	38E−08						
Lta (TNFb/ lymphotoxin A)*	3.8	6.2	110	9E−26	3E−24	Ifng	−2.4	5.9	35	3E−09	8E−08						
Il1b*	2.6	4.5	6.2	0.01	0.03	Tnfsf10 (TRAIL)	−1.6	4.0	19	2E−05	0.0002						
Tnfsf11 (TRANCE)	2.3	6.6	35	3E−09	2E−08	Lif	−1.4	3.6	11	0.0009	0.005						
Csf1 (M-CSF)*	2.3	3.0	11	0.0009	0.003												
Tnfsf10 (TRAIL)	1.1	4.0	9.7	0.002	0.005												

Genes shown are censored at FDR $p \leq 0.05$ and log(2) fold change of ±1 and ordered by log fold change. CPM, counts per million; FDR, false discovery rate; LR, likelihood ratio.
*Genes are differentially expressed in both humans and mice.

OPEN ACCESS CellPress

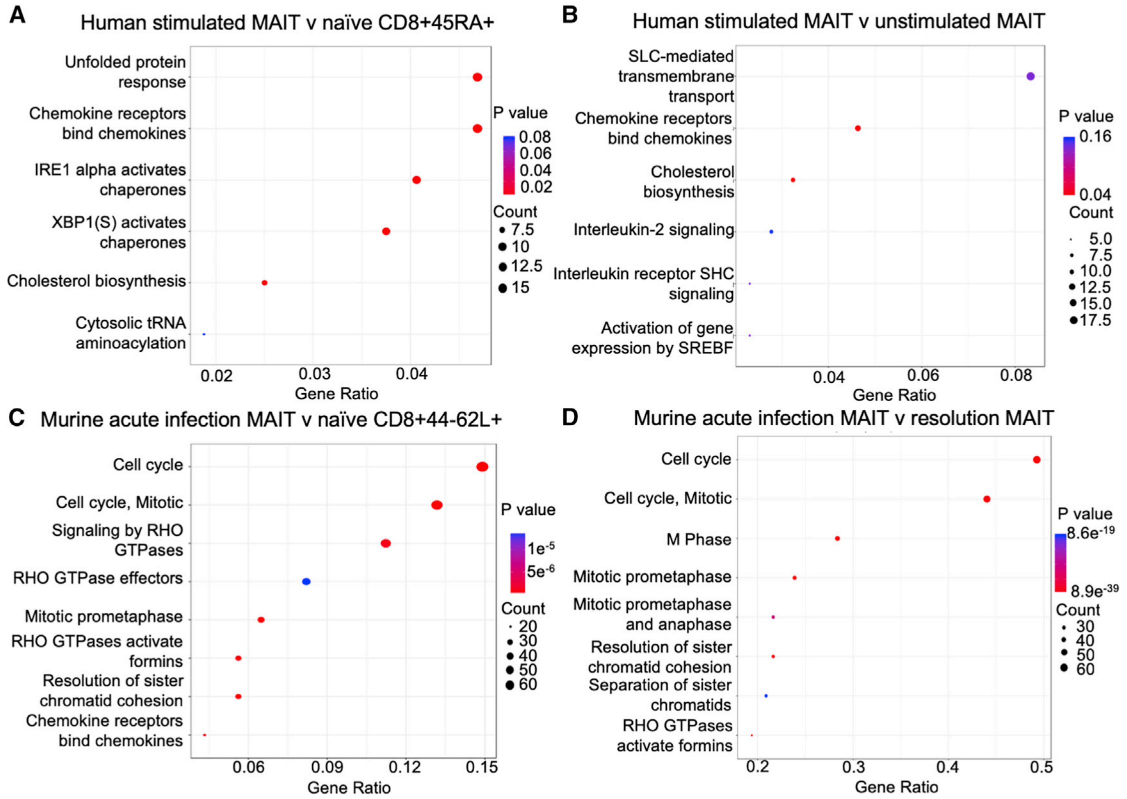

Figure 2. Reactome Pathway Analysis of Activated MAIT Cells

Pathway analysis of human and murine activated MAIT cell transcriptomes.

(A and B) Human peripheral blood 5-OP-RU-stimulated MR1-5-OP-RU-tetramer$^+$ MAIT cells compared with (A) naive CD8$^+$CD45RA$^+$ cells or (B) unstimulated MAIT cells.

(C and D) Murine pulmonary MR1-5-OP-RU-tetramer$^+$ MAIT cells day 7 post infection with *Legionella* were compared with (C) naive CD8$^+$CD44$^-$CD62L$^+$ T cells from uninfected mice or (D) MR1-tetramer$^+$ MAIT cells from mice 12 weeks post infection with *Legionella*.

Plots show the extent to which named pathways from the curated Reactome database are upregulated. Color intensity represents statistical significance of the upregulation, dot size represents the number of genes upregulated in the pathway, x axis represents the proportion of all differentially expressed genes included in the pathway ("gene ratio"). n = 3 biological replicates per group performed once. Pathways were selected using a significance threshold of a log fold change >2 and $p < 0.01$.

iNKT cells by relative upregulation of Plekstrin (Plek), Lyn proto-oncogene (Lyn), SH2 domain containing 1b (Sh2d1b1, a regulator of natural killer cell effector functions), and integrin α X chain protein (Itgax, CD11c). Reactome analysis showed MAIT cells had an upregulation of pathways associated with neutrophil degranulation and pathways associated with cell surface interactions with vasculature. MAIT cells at resolution differed from $\gamma\delta$ T cells most by upregulation of cell surface receptors for IL-18 (Il18r1), vitamin D (Vdr), and leukotriene B4 (Ltb4r1) and in pathways associated with RNA transcription.

MAIT Cells Express a Tissue Repair Transcriptional Profile

Our observation of a distinct cytokine signature after infection resolution suggested that MAIT cells might be capable of performing more diverse functions than a purely pro-inflammatory response to TCR ligation. As observed already, TCR ligation in the absence of a TLR-agonist did not induce proliferation of murine MAIT cells. A wide variety of bacteria, mycobacteria, and yeasts, including many commensal organisms (Kanehisa and Goto, 2000), express the riboflavin biosynthetic pathway and may therefore be a major source of activating MR1 ligands, constitutively, or during breach of a barrier surface. Indeed, MAIT cells require commensal organisms for their expansion (Treiner et al., 2003). A class of skin-homing Tc17 cells specific to commensal flora, which expresses a "tissue repair" gene signature and can accelerate repair of an epithelial wound, has recently been described (Linehan et al., 2018). These cells share several features with non-classical T cells, including the type 17 cytokine profile and restriction by another MHC class 1b antigen presentation molecule H2-M3. Therefore, we asked whether this tissue repair phenotype was a shared transcriptional program in MAIT cells. We used gene set enrichment analysis (GSEA) (Mootha et al., 2003) to compare the expression of this set of tissue repair genes (Linehan et al., 2018) (Table S10) with genes differentially expressed in MAIT cells. Indeed, this gene set was markedly enriched in human MAIT cells after 5-OP-RU stimulation (normalized enrichment score [NES] 1.38; familywise error rate

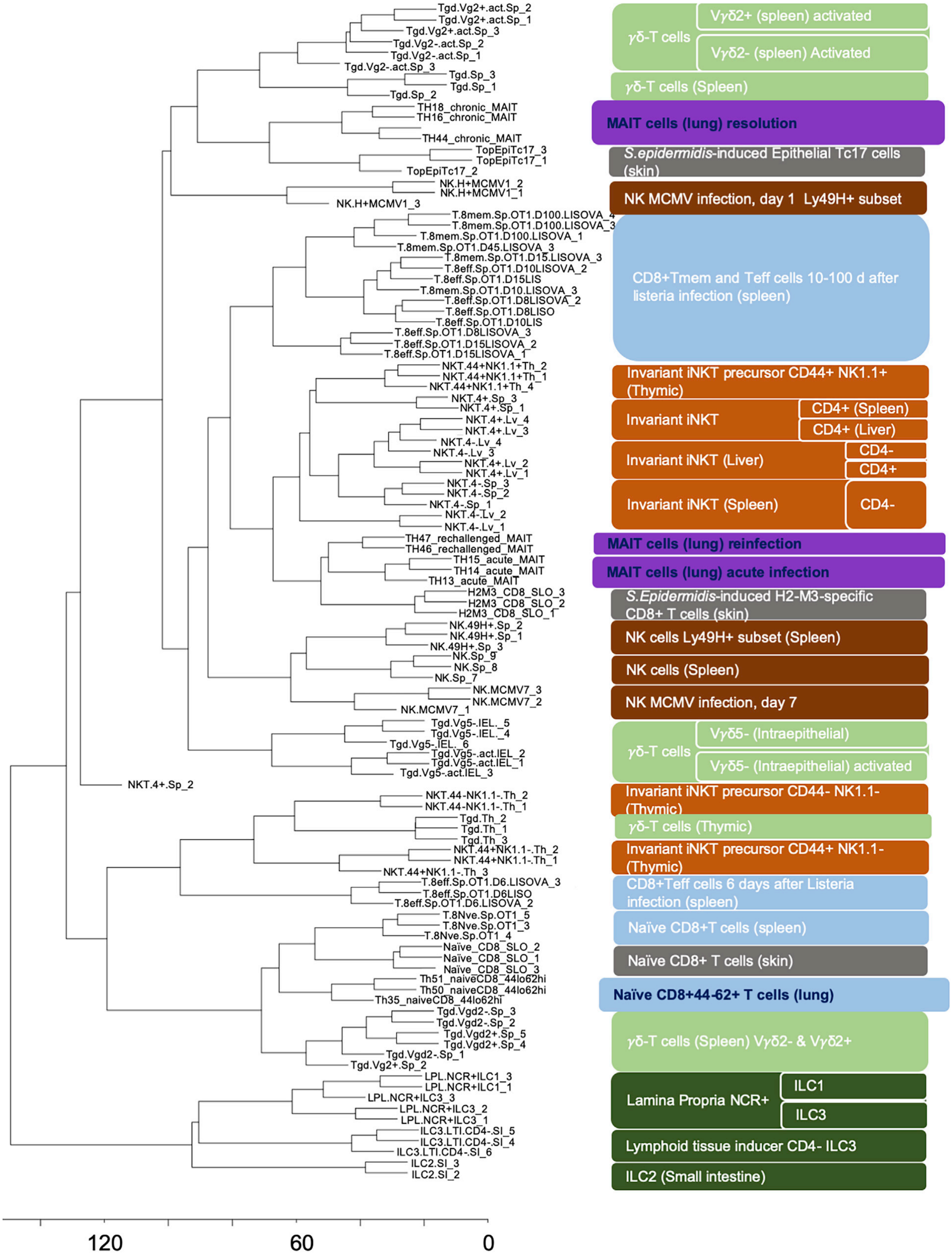

(legend on next page)

OPEN ACCESS
CellPress

[FWER] $p < 0.01$; Figures 4A and 4B; Table 3). By contrast, there was no significant enrichment of this gene signature when the same analysis was performed on published gene expression data (Fernandez et al., 2009) from negatively selected CD3+ T cells stimulated for 24 h with antibody co-stimulation of CD3/CD28 (Figure S6B). Similarly, despite differences in species, time course, and method of MAIT cell activation, the same gene set was even more highly enriched in mice during acute *L. longbeachae* infection (NES 1.38; FWER $p < 0.01$; Figures 4C and 4D; Table 3), with enrichment of 10 genes common to both analyses (TNF, CSF2, HIF1A, FURIN, VEGFB, PTGES2, PDGFB, TGFB1, MMP25, and HMGB1).

Finally, to investigate the extent to which this similarity to H2-M3 restricted T cells was a property specific to MAIT cells, we repeated the previous hierarchical clustering while incorporating published (Linehan et al., 2018) RNA sequencing transcriptomes of three additional cell types: H2-M3 f-MIIINA: H2-M3-tetramer+ CD8+ T cells from mice that had been topically associated with the skin commensal *S. epidermidis* strain NIHLM087, from which the "tissue repair signature" was first identified and that had been activated *in vivo* by peptide injection; CCR6+ skin Tc17 cells obtained from mice that were previously associated with this skin commensal; and additional naive CD8+ T cells from secondary lymphoid organs of uninfected specific-pathogen-free mice (Figure 3, gray lozenges; Table S2). As expected, activated H2-M3-specific CD8+ T cells that had been activated by *in vivo* peptide stimulation clustered immediately adjacent to the MAIT cells from acute infection or reinfection, in which we had observed the tissue repair signature. The skin epithelial cells obtained without recall challenge clustered immediately adjacent to the MAIT cells that were at resolution state, while the additional naive CD8 T cells from secondary lymphoid organs clustered with the other naive CD8+ T cell subsets.

DISCUSSION

Here, we have systematically investigated the requirements for TCR-mediated activation of MAIT cells in mice and we delineated both *ex vivo* in human and *in vivo* in mice the consequences of this activation at a transcriptomic level. Because of their pro-inflammatory cytokine profile (Dusseaux et al., 2011) and specificity for a restricted selection of microbially derived small molecules (Corbett et al., 2014; Kjer-Nielsen et al., 2012), the most immediately apparent function of MAIT cells has hitherto been the early detection of microbes and initiation of an inflammatory host response (Godfrey et al., 2015; Le Bourhis et al., 2013, 2010; Meierovics et al., 2013; Meierovics and Cowley, 2016). Consistent with previous studies, our data confirm MAIT cells' capacity for a strong, rapid pro-inflammatory response. However, in contrast to the similarities between activated MAIT cells and iNKT cells, the close similarity at a transcriptional level of resting murine MAIT cells to $\gamma\delta$ T cells and the discovery of a clear transcriptional signature for tissue repair suggest that MAIT cells potentially have much broader roles in mucosal immunity.

The nature of these roles may depend on the context and nature of the cells' activation. As with iNKT cells (Holzapfel et al., 2014), MAIT cells may be activated either via TCR recognition of ligand presented on MR1 or via cytokines alone, in the absence of a TCR signal, as occurs during respiratory viral infection (Loh et al., 2016; Paquin-Proulx et al., 2018; van Wilgenburg et al., 2018, 2016). As we have observed previously, in the absence of inflammatory cytokines, TCR ligation alone is not sufficient to produce MAIT cell proliferation and activation *in vivo* (Chen et al., 2017). Rather, a second signal is required. *In vitro* in humans, it has been shown that agonists of TLR1, TLR2, and TLR6 can provide this co-stimulus to drive MAIT cell cytokine secretion (Ussher et al., 2016). Consistent with, and extending previous, observations (Chen et al., 2017), we observed here that murine MAIT cells proliferated in response to a different, but similarly restricted, set of TLR agonists: those for TLR3, TLR4, TLR6/2, and TLR9, but not for other TLRs tested. Thus, specific activation of the MAIT TCRs in the context of specific TLR stimulation is sufficient for rapid and robust MAIT cell expansion, which is of similar magnitude and time course to that observed with the *Legionella* model we used for the transcriptomic experiments.

While MAIT cells have been considered pro-inflammatory, our data suggest an entirely distinct function for MAIT cells in tissue repair. In both human and murine datasets, activated MAIT cells highly express a shared gene expression signature with murine H2-M3 restricted commensal-specific Tc17 cells recently reported by Linehan et al., (2018). Using topical skin colonization with a specific clade of *S. epidermidis*, this group demonstrated that specific commensal-derived *N*-formylated peptides presented on H2-M3, another class 1b MHC molecule, could induce tissue-resident Tc17 cells, which provided specific capacity to promote tissue repair and remodeling (MMP25, Furin, PDGFB, TGFB1) and angiogenesis (CSF2, VEGFA, PDGFB) (Linehan et al., 2018). Healing of skin wounds was shown to be accelerated by colonization of H2-M3-sufficient mice with these commensals. A human equivalent of H2-M3 has yet to be identified, but MAIT cells are abundant at barrier sites, allowing close interactions with commensal bacteria possessing an intact riboflavin metabolic pathway. Similar to H2-M3-restricted $CD8^+$ T cells, in this position,

Figure 3. Comparison of Murine MAIT Cell Transcriptomes with Other Cells in Immunological Genome Project Dataset and with Commensal-Induced H2-M3-Restricted T Cells

Hierarchical clustering was used to compare transcriptomes of murine pulmonary MAIT cells or naive $CD8^+CD44^-CD62L^+$ cells in this study with 88 other cell types deposited in the Immunological Genome Project (ImmGen) database and three cell types selected from Linehan et al., (2018) (gray lozenges). Figure shows a dendrogram (left), ImmGen identifiers (middle), and the full name of each cell type (right). Further details on cell types are presented in Table S2. ImmGen samples are identified in white lettering. Samples from the current study are identified in black lettering, with extended lozenges. Each cell type is represented by 2 to 3 replicates, identified by a numerical suffix, of which each replicate ("batch") represents pooled tissue from three animals. Cell types are color coded: invariant natural killer T cells (iNKT, orange), natural killer (NK) cells (brown), $\gamma\delta$ T cells (light green), innate lymphoid cells (ILC, dark green), conventional CD8 T cells (blue), and MAIT cells (purple). CD, clonal designation; NCR, NK cell receptor; Teff, effector T cell; Tmem, memory T cell.

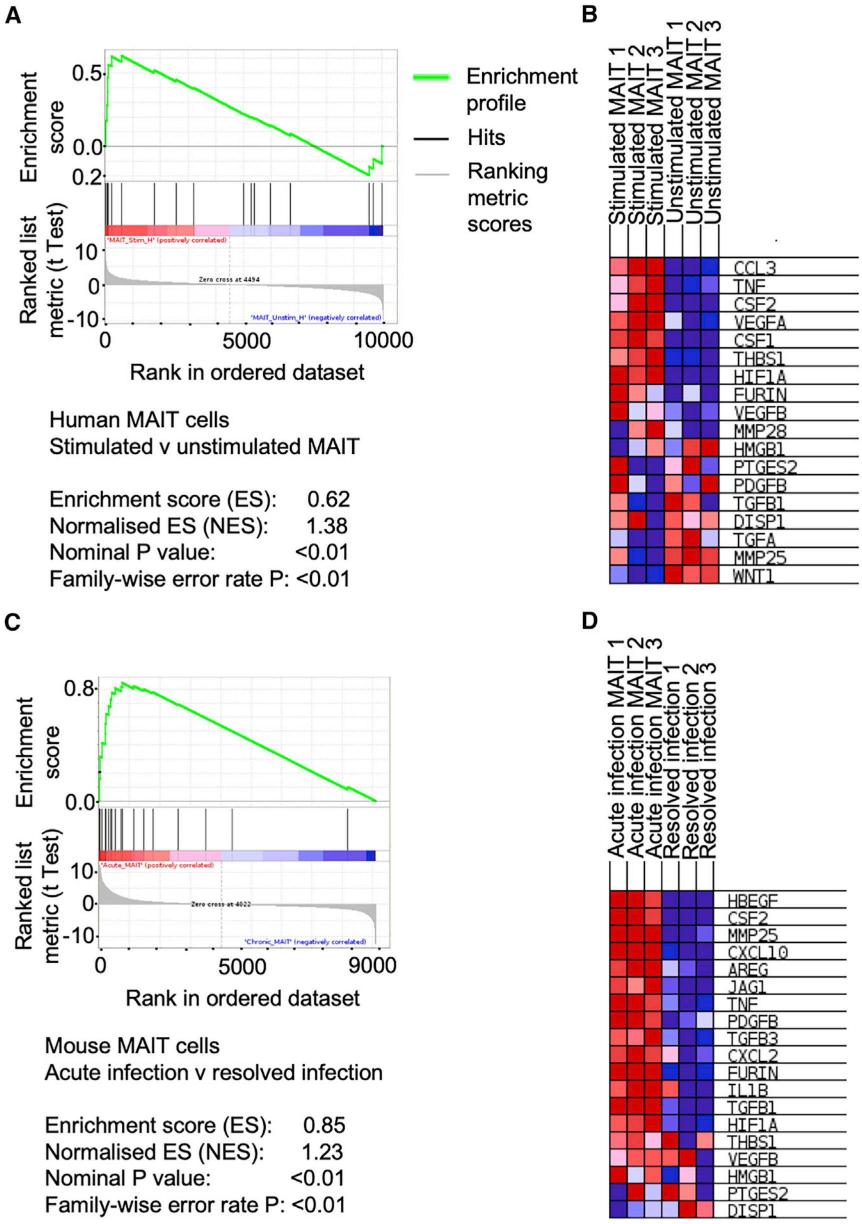

Figure 4. Gene Set Enrichment Analysis for Tissue Repair Gene Signature in Human and Murine MAIT Cells

(A–D) Gene set enrichment analysis (GSEA) was used to determine potential enrichment of a tissue repair signature (Linehan et al., 2018) in gene expression profiles from human (A and B) and murine (C and D) MAIT cells.

(A) GSEA summary plots for 5-OP-RU-stimulated human peripheral blood MAIT cells compared with unstimulated MAIT cells. The gene set is highly enriched: enrichment score (ES) = 0.62; normalized enrichment score (NES) = 1.38; nominal p value < 0.01; familywise error rate (FWER) p value < 0.01.

(B) Heatmap of expression of leading-edge subset genes within the gene set (red, highest expression; blue, lowest).

(C) GSEA summary plots for murine pulmonary MAIT cells 7 days post i.n. *L. longbeachae* infection (“Acute infection”), compared with MAIT cells 12 weeks post infection (“Resolved infection”). The gene set is highly enriched: enrichment score (ES) = 0.85; normalized enrichment score (NES) = 1.23; nominal p value < 0.01; familywise error rate (FWER) p value < 0.01.

(D) Heatmap of expression of leading-edge subset genes within the gene set (red, highest expression; blue, lowest). n = 3 biological replicates per group performed once.

MAIT cells are poised to maintain tissue homeostasis in the presence of commensals, thereby limiting inflammation and associated tissue injury (Klenerman and Ogg, 2018). We did not observe enrichment of this tissue repair signature in published gene expression data from TCR-stimulated CD3+ T cells, suggesting this functionality is not a common property of most T cells, but is restricted to only certain T cell subsets. Indeed, when TCR-activated commensal-specific H2-M3-restricted T cells were incorporated into our hierarchical cluster analysis of 100 other lymphocyte subsets, they clustered very closely with the activated MAIT cell subsets, while skin Tc17 cells without activation clustered most closely with MAIT cells at the resolution of infection. These findings provide additional evidence to suggest strong functional similarities between H2-M3-restricted Tc17 cells and the MAIT cell subset specifically. Furthermore, these data are consistent with similar findings of this same tissue repair signature observed by gene set enrichment analysis of MAIT cells when activated by TCR triggering, but not observed in the context of cytokine-mediated activation (Lamichhane et al., 2019; Leng et al., 2019). These findings might also explain the increased gut permeability observed in *Mr1*$^{-/-}$ NOD mice compared with *Mr1*$^{+/-}$ NOD littermates, which suggested a protective role for MAIT cells for maintaining gut homeostasis (Rouxel et al., 2017). It has been speculated this might be mediated by IL-17A and IL-22 (Rouxel et al., 2017), which are both important in intestinal homeostasis (Dudakov et al., 2015; Lee et al., 2015) and, in the case of IL-22, induction of protective mucus-producing goblet cells (Sugimoto et al., 2008). In our dataset, both cytokines were strongly upregulated in activated murine MAIT cells. Thus, during mucosal damage, riboflavin-synthesizing pathogens and commensal organisms might provide both the MAIT cell activation to induce the necessary inflammatory response to ensure bacterial clearance and the signals necessary to accelerate healing of the wound. After a successful clearance of infection, or barrier repair, the subsequent reduction in MR1-Ag presentation would ensure this signal declined.

Table 3. Gene Set Enrichment Analysis for Tissue Repair Set

Human Stimulated MAIT versus Unstimulated MAIT					Murine Acute Infection versus Resolved Infection				
Gene	Rank in Gene List	Rank Metric Score	Running Enrichment Score	Core Enrichment	Gene	Rank in Gene List	Rank Metric Score	Running Enrichment Score	Core Enrichment
CCL3	16	7.566	0.180	yes	HBEGF	5	12.242	0.162	yes
TNF*	63	4.421	0.282	yes	CSF2*	33	11.994	0.319	yes
CSF2*	85	4.012	0.376	yes	MMP25*	97	7.929	0.417	yes
VEGFA	87	3.990	0.471	yes	CXCL10	210	5.741	0.481	yes
CSF1	105	3.700	0.559	yes	AREG	216	5.579	0.555	yes
THBS1	226	2.790	0.614	yes	JAG1	234	5.371	0.625	yes
HIF1A*	594	1.725	0.618	yes	TNF*	311	4.744	0.679	yes
FURIN*	1,763	0.836	0.522	no	PDGFB*	375	4.226	0.729	yes
VEGFB*	2,560	0.539	0.456	no	TGFB3	414	3.921	0.777	yes
MMP28	3,207	0.339	0.399	no	CXCL2	529	3.295	0.808	yes
HMGB1*	5,005	−0.120	0.223	no	FURIN*	721	2.474	0.820	yes
PTGES2*	5,279	−0.189	0.200	no	IL1B	772	2.296	0.845	yes
PDGFB*	5,399	−0.218	0.194	no	TGFB1*	1,134	1.427	0.824	no
TGFB1*	5,973	−0.374	0.146	no	HIF1A*	1,464	1.033	0.802	no
DISP1	6,691	−0.589	0.088	no	THBS1	1,761	0.801	0.780	no
TGFA	9,539	−2.469	−0.136	no	VEGFB*	2,604	0.398	0.692	no
MMP25*	9,697	−2.794	−0.085	no	HMGB1*	3,518	0.118	0.594	no
WNT1	10,012	−4.987	0.003	no	PTGES2*	4,382	−0.084	0.500	no
					DISP1	8,193	−1.502	0.101	no

Genes set enrichment analysis (GSEA) was used to determine potential enrichment of a tissue repair signature (Linehan et al., 2018) in gene expression profiles of 5-OP-RU-stimulated human peripheral blood MAIT cells compared with unstimulated MAIT cells (left) and of murine pulmonary MAIT cells 7 days post i.n. *L. longbeachae* infection, compared with MAIT cells 12 weeks post infection (right). Genes are ordered by their position in the list of genes ranked by their normalized enrichment score (ES). Running enrichment score: ES at this point in the ranked list of genes. Core enrichment genes contribute to the leading-edge subset of genes that contribute most to the enrichment result.
*Genes are also significant in the equivalent analysis for murine MAIT cells.

Commensals might drive the MR1-MAIT cell axis in other ways. MAIT cell expansion requires exposure to a commensal microbiome (Treiner et al., 2003). Furthermore, commensal microbes have been implicated in enhancing host immunity against pathogens in the respiratory tract. In mice, a Nod2-mediated IL-17A response to upper-respiratory tract commensals enhanced CSF2 (GM-CSF) to promote bacterial killing and clearance by alveolar macrophages (Brown et al., 2017). The strong upregulation of CSF2 (GM-CSF) we observed following TCR stimulation in human and murine MAIT cells would be beneficial in the clearance of pathogenic microorganisms that have crossed the mucosal barrier. During tissue homeostasis, commensal-derived MR1 signals might drive lower-level, constitutive expression of GM-CSF needed to maintain alveolar macrophages in a pathogen-responsive state (Brown et al., 2017).

Another novel, prominent feature of MAIT cell activation in both humans and mice was the marked expression of the IL-6 family cytokine leukemia inhibitory factor (LIF). Consistent with our findings of a MAIT cell tissue repair signature, the LIF has been found to protect against epithelial damage in murine models of pneumonia (Quinton et al., 2012). The LIF is significantly induced during pneumonia and can reduce lung epithelial cell death, promoting the expression of tissue-protective genes essential to lung regeneration and repair and increased mucosal barrier integrity.

A unique feature of our transcriptomic dataset is that in a single experiment, we were able to analyze MAIT cells from two different species obtained from two different tissues, using different contexts of activation, and yet we observed that the transcriptomic profiles of these MAIT cells were in fact very similar. Thus, the distinctive, common properties of MAIT cells predominate over differences between these cells that might be observed in different contexts. Again, this underlines a strong conservation of functions likely driven by a consistent role in mucosal immunology.

Nonetheless, we also observed some differences between the human and murine datasets. In particular, the Reactome pathway analysis showed a very prominent gene signature for cell cycle and mitotic pathways not observed in the parallel human pathway analysis. This is unlikely to be due to differences in species, but rather to the different context of cell activation. Several previous studies have shown that to achieve maximal, sustained MAIT cell activation requires cytokine signals in addition to TCR triggering. These signals have been shown to include IL-12, IL-18 (Ussher et al., 2014), IL-15, and type 1 interferons, both *in vitro* (van Wilgenburg et al., 2016) and *in vivo* (van Wilgenburg et al., 2018). While IL-12 and IL-18 are particularly important for inducing cytokine production from MAIT cells (Ussher et al., 2014; Leng et al., 2019), the induction of MAIT cell proliferation

appears to be more dependent on IL-15 (Kurioka et al., 2015). In the current study, such synergistic cytokine signals would have been present in the *in vivo* TLR/5-OP-RU or *Legionella* infection models, but not in the human *in vitro* stimulation. In addition, the time courses of these two models were very different: 6 h for the *in vitro* stimulation, compared with 7 days *in vivo*, allowing much greater time for cell proliferation. It is therefore more striking that despite these two important differences, the consistent tissue repair signature was apparent in both models.

Given the wide diversity of conventional and non-classical T cells now recognized (Godfrey et al., 2015), many of which share common transcriptional programs (Cohen et al., 2013; Kurioka et al., 2018), we applied a comparative approach (Lee et al., 2016) to analyze the phenotype of MAIT cells, overcoming significant methodological hurdles to compare our RNA sequencing data directly with older microarray expression data in the ImmGen dataset (Heng et al., 2008). Activated MAIT cells were most similar to activated invariant iNKT cells, as might be expected from the similarities in surface markers and functional phenotype (Eckle et al., 2014; Godfrey et al., 2015; Porcelli et al., 1993). This is likely related to shared transcriptional signatures controlled by common transcription factors, not least that that has been described for promyelocytic leukemia zinc finger (PLZF), which defines a distinct surface phenotype and functional capacity in $CD161^+$ NK cells, iNKT cells, and MAIT cells (Kurioka et al., 2018). However, it is interesting that in their resting state, MAIT cells more closely resembled splenic $\gamma\delta$ T cells. While MAIT cells differed from $\gamma\delta$ T cells by higher expression of specific cell surface receptors—notably the IL-18 receptor, recognized as a classic MAIT cell marker (Dusseaux et al., 2011) and the vitamin D receptor (Vdr), consistent with their postulated modulation by vitamin D (Hinks et al., 2015)—resting MAIT cells differed more from iNKT cells. These differences were dominated by upregulation of pathways associated with degranulation and pathways associated with cell surface interactions with vasculature, suggesting that after the resolution of infection, MAIT cells may retain more capacity for transmigration and for cytotoxic degranulation than iNKT cells, although this comparison may also have been influenced by the different tissue origins of our MAIT cell data (pulmonary) and the iNKT cell data from ImmGen (spleen and liver). Unlike MAIT and iNKT cells, most $\gamma\delta$ T cells are not constrained by a specific MHC restriction (Chien et al., 2014; Vantourout and Hayday, 2013); rather, they have different functional profiles associated with the usage of different TCR V gene segments. Depending on the $V\gamma$ subset, $\gamma\delta$ T cells recognize a diverse range of small microbial metabolites, lipids, self-antigens, and stress-induced proteins and may display a range of functions associated with inflammation, immunoregulation, cytotoxicity, antigen presentation (Godfrey et al., 2015), and promotion of tissue repair (Havran and Jameson, 2010; Nielsen et al., 2017). In the absence of the TCR-/TLR-mediated activation, MAIT cells may be fulfilling a different, perhaps homeostatic, function. Indeed, we were able to investigate what this might be by analyzing the transcriptome of MAIT cells in their resting state, outside the context of inflammation. After the resolution of infection, Tnfsf18 (GITRL) is the most strongly upregulated cytokine. The function of GITRL is context dependent, but under resting, non-inflammatory conditions it can negatively regulate NK cells and maintain or expand regulatory T cells' conditions (Clouthier and Watts, 2014). Other immunoregulatory cytokines were also upregulated: Tnfsf11 (TRANCE, RANKL) was identified in a commensal-derived immunoregulatory signature (Linehan et al., 2018), while IL-15 can inhibit T cell apoptosis to maintain memory T cell survival (Malamut et al., 2010). Together, these data implicate resting MAIT cells in potentially significant immunoregulatory roles.

In summary, our analysis of TCR-activated MAIT cells demonstrates a pronounced conservation of functions and gene expression profiles between human and murine cells and suggests that beyond type 17/type 1 pro-inflammatory responses to invading microbial pathogens, MAIT cells have the capacity to contribute to immunoregulatory and tissue repair roles likely to be essential for maintaining the integrity of mucosal barrier surfaces in health and disease.

STAR★METHODS

Detailed methods are provided in the online version of this paper and include the following:

- KEY RESOURCES TABLE
- LEAD CONTACT AND MATERIALS AVAILABILITY
- EXPERIMENTAL MODEL AND SUBJECT DETAILS
  - Mice
  - Human volunteers
  - Bacterial strains
- METHOD DETAILS
  - Compounds, immunogens and tetramers
  - *In vivo* infection
  - Tissue processing
  - Determination of bacterial counts in infected lungs
  - Antibodies flow cytometry and cell sorting
  - RNA sequencing
- QUANTIFICATION AND STATISTICAL ANALYSIS
  - Quality control and analysis of RNA sequencing data
  - Standard statistical analysis
- DATA AND CODE AVAILABILITY

SUPPLEMENTAL INFORMATION

Supplemental Information can be found online at https://doi.org/10.1016/j.celrep.2019.07.039.

ACKNOWLEDGMENTS

This work was funded by grants to T.S.C.H. from the Wellcome Trust (104553/z/14/z and 211050/Z/18/z). The work was supported by the National Health and Medical Research Council of Australia (NHMRC) program grants 1113293, 1071916, 1016629, and 606788 and project grant 1120467. A.J.C. is supported by an ARC Future Fellowship. S.B.G. is supported by an ARC DECRA Fellowship. P.K. was supported by an NIHR Senior Fellowship, Oxford Martin School, and the Wellcome Trust (WT109965MA). This work benefitted from data assembled by the ImmGen consortium (Heng et al., 2008). We are grateful to Dr. Brendan Russ and Dr. Linda Wakim for assistance and suggestions for experimental design; Dr. Ama Essilfie, Prof. Richard Strugnell, Frances Oppodisam, Jennifer Davies, Prof. Roy Robbins-Browne, Prof. Kenneth Beagley, and Dr. Hayley Newton for bacterial strains; Prof. David Jackson for Pam2Cys; Dr. Jeffrey Mak for MR1 ligands; Dr. Vanta Jameson, Mr. Josh Kie at the Flow Cytometry Facilities at the Melbourne Brain Centre and the

Peter Doherty Institute; and Kym Pham and Karey Cheong at the Melbourne Translational Genomic Platform.

AUTHOR CONTRIBUTIONS

T.S.C.H., T.J.P., L.K., and B.S.M. performed the experiments. T.S.C.H., M.O., E.M., A.K., and M.J. analyzed the data. T.S.C.H., J.M., Z.C., A.J.C., S.J.T., and P.K. designed the experiments and managed the study. T.S.C.H., M.J., A.J.C., Z.C., J.M., and P.K. conceived the work and wrote the manuscript, which was revised and approved by all authors.

DECLARATION OF INTERESTS

Z.C., J.M., and A.J.C. are inventors on patents describing MR1 tetramers and MR1 ligands. The other authors declare no conflicting interests.

Received: December 7, 2018
Revised: April 17, 2019
Accepted: July 12, 2019
Published: September 17, 2019

REFERENCES

Brown, R.L., Sequeira, R.P., and Clarke, T.B. (2017). The microbiota protects against respiratory infection via GM-CSF signaling. Nat. Commun. *8*, 1512.

Cazalet, C., Gomez-Valero, L., Rusniok, C., Lomma, M., Dervins-Ravault, D., Newton, H.J., Sansom, F.M., Jarraud, S., Zidane, N., Ma, L., et al. (2010). Analysis of the Legionella longbeachae genome and transcriptome uncovers unique strategies to cause Legionnaires' disease. PLoS Genet. *6*, e1000851.

Chen, Z., Wang, H., D'Souza, C., Sun, S., Kostenko, L., Eckle, S.B., Meehan, B.S., Jackson, D.C., Strugnell, R.A., Cao, H., et al. (2017). Mucosal-associated invariant T-cell activation and accumulation after in vivo infection depends on microbial riboflavin synthesis and co-stimulatory signals. Mucosal Immunol. *10*, 58–68.

Chien, Y.H., Meyer, C., and Bonneville, M. (2014). γδ T cells: first line of defense and beyond. Annu. Rev. Immunol. *32*, 121–155.

Chua, W.J., Truscott, S.M., Eickhoff, C.S., Blazevic, A., Hoft, D.F., and Hansen, T.H. (2012). Polyclonal mucosa-associated invariant T cells have unique innate functions in bacterial infection. Infect. Immun. *80*, 3256–3267.

Clouthier, D.L., and Watts, T.H. (2014). Cell-specific and context-dependent effects of GITR in cancer, autoimmunity, and infection. Cytokine Growth Factor Rev. *25*, 91–106.

Cohen, N.R., Brennan, P.J., Shay, T., Watts, G.F., Brigl, M., Kang, J., and Brenner, M.B.; ImmGen Project Consortium (2013). Shared and distinct transcriptional programs underlie the hybrid nature of iNKT cells. Nat. Immunol. *14*, 90–99.

Corbett, A.J., Eckle, S.B., Birkinshaw, R.W., Liu, L., Patel, O., Mahony, J., Chen, Z., Reantragoon, R., Meehan, B., Cao, H., et al. (2014). T-cell activation by transitory neo-antigens derived from distinct microbial pathways. Nature *509*, 361–365.

Dobin, A., Davis, C.A., Schlesinger, F., Drenkow, J., Zaleski, C., Jha, S., Batut, P., Chaisson, M., and Gingeras, T.R. (2013). STAR: ultrafast universal RNA-seq aligner. Bioinformatics *29*, 15–21.

Dudakov, J.A., Hanash, A.M., and van den Brink, M.R. (2015). Interleukin-22: immunobiology and pathology. Annu. Rev. Immunol. *33*, 747–785.

Dusseaux, M., Martin, E., Serriari, N., Péguillet, I., Premel, V., Louis, D., Milder, M., Le Bourhis, L., Soudais, C., Treiner, E., and Lantz, O. (2011). Human MAIT cells are xenobiotic-resistant, tissue-targeted, CD161hi IL-17-secreting T cells. Blood *117*, 1250–1259.

Eckle, S.B., Birkinshaw, R.W., Kostenko, L., Corbett, A.J., McWilliam, H.E., Reantragoon, R., Chen, Z., Gherardin, N.A., Beddoe, T., Liu, L., et al. (2014). A molecular basis underpinning the T cell receptor heterogeneity of mucosal-associated invariant T cells. J. Exp. Med. *211*, 1585–1600.

Eckle, S.B., Corbett, A.J., Keller, A.N., Chen, Z., Godfrey, D.I., Liu, L., Mak, J.Y., Fairlie, D.P., Rossjohn, J., and McCluskey, J. (2015). Recognition of Vitamin B Precursors and Byproducts by Mucosal Associated Invariant T Cells. J. Biol. Chem. *290*, 30204–30211.

Fabregat, A., Jupe, S., Matthews, L., Sidiropoulos, K., Gillespie, M., Garapati, P., Haw, R., Jassal, B., Korninger, F., May, B., et al. (2018). The Reactome Pathway Knowledgebase. Nucleic Acids Res. *46* (*D1*), D649–D655.

Fernandez, D.R., Telarico, T., Bonilla, E., Li, Q., Banerjee, S., Middleton, F.A., Phillips, P.E., Crow, M.K., Oess, S., Muller-Esterl, W., and Perl, A. (2009). Activation of mammalian target of rapamycin controls the loss of TCRzeta in lupus T cells through HRES-1/Rab4-regulated lysosomal degradation. J. Immunol. *182*, 2063–2073.

Gentleman, R.C., Carey, V.J., Bates, D.M., Bolstad, B., Dettling, M., Dudoit, S., Ellis, B., Gautier, L., Ge, Y., Gentry, J., et al. (2004). Bioconductor: open software development for computational biology and bioinformatics. Genome Biol. *5*, R80.

Godfrey, D.I., Uldrich, A.P., McCluskey, J., Rossjohn, J., and Moody, D.B. (2015). The burgeoning family of unconventional T cells. Nat. Immunol. *16*, 1114–1123.

Gold, M.C., Eid, T., Smyk-Pearson, S., Eberling, Y., Swarbrick, G.M., Langley, S.M., Streeter, P.R., Lewinsohn, D.A., and Lewinsohn, D.M. (2013). Human thymic MR1-restricted MAIT cells are innate pathogen-reactive effectors that adapt following thymic egress. Mucosal Immunol. *6*, 35–44.

Havran, W.L., and Jameson, J.M. (2010). Epidermal T cells and wound healing. J. Immunol. *184*, 5423–5428.

Heng, T.S., and Painter, M.W.; Immunological Genome Project Consortium (2008). The Immunological Genome Project: networks of gene expression in immune cells. Nat. Immunol. *9*, 1091–1094.

Hinks, T.S., Zhou, X., Staples, K.J., Dimitrov, B.D., Manta, A., Petrossian, T., Lum, P.Y., Smith, C.G., Ward, J.A., Howarth, P.H., et al. (2015). Innate and adaptive T cells in asthmatic patients: Relationship to severity and disease mechanisms. J. Allergy Clin. Immunol. *136*, 323–333.

Holzapfel, K.L., Tyznik, A.J., Kronenberg, M., and Hogquist, K.A. (2014). Antigen-dependent versus -independent activation of invariant NKT cells during infection. J. Immunol. *192*, 5490–5498.

Jabri, B., and Abadie, V. (2015). IL-15 functions as a danger signal to regulate tissue-resident T cells and tissue destruction. Nat. Rev. Immunol. *15*, 771–783.

Kanehisa, M., and Goto, S. (2000). KEGG: kyoto encyclopedia of genes and genomes. Nucleic Acids Res. *28*, 27–30.

Kjer-Nielsen, L., Patel, O., Corbett, A.J., Le Nours, J., Meehan, B., Liu, L., Bhati, M., Chen, Z., Kostenko, L., Reantragoon, R., et al. (2012). MR1 presents microbial vitamin B metabolites to MAIT cells. Nature *491*, 717–723.

Klenerman, P., and Ogg, G. (2018). Killer T cells show their kinder side. Nature *555*, 594–595.

Kurioka, A., Ussher, J.E., Cosgrove, C., Clough, C., Fergusson, J.R., Smith, K., Kang, Y.H., Walker, L.J., Hansen, T.H., Willberg, C.B., and Klenerman, P. (2015). MAIT cells are licensed through granzyme exchange to kill bacterially sensitized targets. Mucosal Immunol. *8*, 429–440.

Kurioka, A., Cosgrove, C., Simoni, Y., van Wilgenburg, B., Geremia, A., Björkander, S., Sverremark-Ekström, E., Thurnheer, C., Günthard, H.F., Khanna, N., et al.; Swiss HIV Cohort Study; Oxford IBD Cohort Investigators (2018). CD161 Defines a Functionally Distinct Subset of Pro-Inflammatory Natural Killer Cells. Front. Immunol. *9*, 486.

Lamichhane, R., Schneider, M., de la Harpe, S.M., Harrop, T.W.R., Hannawat, R.F., Dearden, P.K., Kirman, J.R., Tyndall, J.D., Vernall, A.J., and Ussher, J.E. (2019). TCR- or Cytokine-Activated CD8+ Mucosal-Associated Invariant T Cells Are Rapid Polyfunctional Effectors That Can Coordinate Immune Responses. Cell Rep. *28*, Published online September 17, 2019. https://doi.org/10.1016/j.celrep.2019.08.054.

Le Bourhis, L., Martin, E., Péguillet, I., Guihot, A., Froux, N., Coré, M., Lévy, E., Dusseaux, M., Meyssonnier, V., Premel, V., et al. (2010). Antimicrobial activity of mucosal-associated invariant T cells. Nat. Immunol. *11*, 701–708.

Le Bourhis, L., Dusseaux, M., Bohineust, A., Bessoles, S., Martin, E., Premel, V., Coré, M., Sleurs, D., Serriari, N.E., Treiner, E., et al. (2013). MAIT cells detect and efficiently lyse bacterially-infected epithelial cells. PLoS Pathog. *9*, e1003681.

Lee, J.S., Tato, C.M., Joyce-Shaikh, B., Gulen, M.F., Cayatte, C., Chen, Y., Blumenschein, W.M., Judo, M., Ayanoglu, G., McClanahan, T.K., et al. (2015). Interleukin-23-Independent IL-17 Production Regulates Intestinal Epithelial Permeability. Immunity *43*, 727–738.

Lee, Y.J., Starrett, G.J., Lee, S.T., Yang, R., Henzler, C.M., Jameson, S.C., and Hogquist, K.A. (2016). Lineage-Specific Effector Signatures of Invariant NKT Cells Are Shared amongst $\gamma\delta$ T, Innate Lymphoid, and Th Cells. J. Immunol. *197*, 1460–1470.

Leek, J.T., Johnson, W.E., Parker, H.S., Jaffe, A.E., and Storey, J.D. (2012). The sva package for removing batch effects and other unwanted variation in high-throughput experiments. Bioinformatics *28*, 882–883.

Leng, T., Akther, H.D., Hackstein, C.-P., Powell, K., King, T., Friedrich, M., Christoforidou, Z., McCuaig, S., Neyazi, M., Arancibia-Cárcamo, C.V., et al. (2019). TCR and Inflammatory Signals Tune Human MAIT Cells to Exert Specific Tissue Repair and Effector Functions. Cell Rep. *28*, 3077–3091.

Lepore, M., Kalinichenko, A., Colone, A., Paleja, B., Singhal, A., Tschumi, A., Lee, B., Poidinger, M., Zolezzi, F., Quagliata, L., et al. (2014). Parallel T-cell cloning and deep sequencing of human MAIT cells reveal stable oligoclonal TCRβ repertoire. Nat. Commun. *5*, 3866.

Liao, Y., Smyth, G.K., and Shi, W. (2013). The Subread aligner: fast, accurate and scalable read mapping by seed-and-vote. Nucleic Acids Res. *41*, e108.

Linehan, J.L., Harrison, O.J., Han, S.J., Byrd, A.L., Vujkovic-Cvijin, I., Villarino, A.V., Sen, S.K., Shaik, J., Smelkinson, M., Tamoutounour, S., et al. (2018). Non-classical Immunity Controls Microbiota Impact on Skin Immunity and Tissue Repair. Cell *172*, 784–796.e718.

Loh, L., Wang, Z., Sant, S., Koutsakos, M., Jegaskanda, S., Corbett, A.J., Liu, L., Fairlie, D.P., Crowe, J., Rossjohn, J., et al. (2016). Human mucosal-associated invariant T cells contribute to antiviral influenza immunity via IL-18-dependent activation. Proc. Natl. Acad. Sci. USA *113*, 10133–10138.

Maggio, S., Takeda, K., Stark, F., Meierovics, A.I., Yabe, I., and Cowley, S.C. (2015). Control of Francisella tularensis Intracellular Growth by Pulmonary Epithelial Cells. PLoS ONE *10*, e0138565.

Maglott, D., Ostell, J., Pruitt, K.D., and Tatusova, T. (2011). Entrez Gene: gene-centered information at NCBI. Nucleic Acids Res. *39*, D52–D57.

Mak, J.Y., Xu, W., Reid, R.C., Corbett, A.J., Meehan, B.S., Wang, H., Chen, Z., Rossjohn, J., McCluskey, J., Liu, L., and Fairlie, D.P. (2017). Stabilizing short-lived Schiff base derivatives of 5-aminouracils that activate mucosal-associated invariant T cells. Nat. Commun. *8*, 14599.

Malamut, G., El Machhour, R., Montcuquet, N., Martin-Lannerée, S., Dusanter-Fourt, I., Verkarre, V., Mention, J.J., Rahmi, G., Kiyono, H., Butz, E.A., et al. (2010). IL-15 triggers an antiapoptotic pathway in human intraepithelial lymphocytes that is a potential new target in celiac disease-associated inflammation and lymphomagenesis. J. Clin. Invest. *120*, 2131–2143.

Meierovics, A.I., and Cowley, S.C. (2016). MAIT cells promote inflammatory monocyte differentiation into dendritic cells during pulmonary intracellular infection. J. Exp. Med. *213*, 2793–2809.

Meierovics, A., Yankelevich, W.J., and Cowley, S.C. (2013). MAIT cells are critical for optimal mucosal immune responses during in vivo pulmonary bacterial infection. Proc. Natl. Acad. Sci. USA *110*, E3119–E3128.

Mootha, V.K., Lindgren, C.M., Eriksson, K.F., Subramanian, A., Sihag, S., Lehar, J., Puigserver, P., Carlsson, E., Ridderstråle, M., Laurila, E., et al. (2003). PGC-1alpha-responsive genes involved in oxidative phosphorylation are coordinately downregulated in human diabetes. Nat. Genet. *34*, 267–273.

Nielsen, M.M., Witherden, D.A., and Havran, W.L. (2017). $\gamma\delta$ T cells in homeostasis and host defence of epithelial barrier tissues. Nat. Rev. Immunol. *17*, 733–745.

Paquin-Proulx, D., Avelino-Silva, V.I., Santos, B.A.N., Silveira Barsotti, N., Siroma, F., Fernandes Ramos, J., Coracini Tonacio, A., Song, A., Maestri, A., Barros Cerqueira, N., et al. (2018). MAIT cells are activated in acute Dengue virus infection and after in vitro Zika virus infection. PLoS Negl. Trop. Dis. *12*, e0006154.

Patel, O., Kjer-Nielsen, L., Le Nours, J., Eckle, S.B., Birkinshaw, R., Beddoe, T., Corbett, A.J., Liu, L., Miles, J.J., Meehan, B., et al. (2013). Recognition of vitamin B metabolites by mucosal-associated invariant T cells. Nat. Commun. *4*, 2142.

Porcelli, S., Yockey, C.E., Brenner, M.B., and Balk, S.P. (1993). Analysis of T cell antigen receptor (TCR) expression by human peripheral blood CD4-8-alpha/beta T cells demonstrates preferential use of several V beta genes and an invariant TCR alpha chain. J. Exp. Med. *178*, 1–16.

Quinton, L.J., Mizgerd, J.P., Hilliard, K.L., Jones, M.R., Kwon, C.Y., and Allen, E. (2012). Leukemia inhibitory factor signaling is required for lung protection during pneumonia. J. Immunol. *188*, 6300–6308.

Rahimpour, A., Koay, H.F., Enders, A., Clanchy, R., Eckle, S.B., Meehan, B., Chen, Z., Whittle, B., Liu, L., Fairlie, D.P., et al. (2015). Identification of phenotypically and functionally heterogeneous mouse mucosal-associated invariant T cells using MR1 tetramers. J. Exp. Med. *212*, 1095–1108.

Reantragoon, R., Corbett, A.J., Sakala, I.G., Gherardin, N.A., Furness, J.B., Chen, Z., Eckle, S.B., Uldrich, A.P., Birkinshaw, R.W., Patel, O., et al. (2013). Antigen-loaded MR1 tetramers define T cell receptor heterogeneity in mucosal-associated invariant T cells. J. Exp. Med. *210*, 2305–2320.

Robinson, M.D., McCarthy, D.J., and Smyth, G.K. (2010). edgeR: a Bioconductor package for differential expression analysis of digital gene expression data. Bioinformatics *26*, 139–140.

Rouxel, O., Da Silva, J., Beaudoin, L., Nel, I., Tard, C., Cagninacci, L., Kiaf, B., Oshima, M., Diedisheim, M., Salou, M., et al. (2017). Cytotoxic and regulatory roles of mucosal-associated invariant T cells in type 1 diabetes. Nat. Immunol. *18*, 1321–1331.

Smyth, G.K. (2016). limma: Linear Models for Microarray Data. In Bioinformatics and Computational Biology Solutions Using R and Bioconductor, R. Gentleman, V.J. Carey, W. Huber, R.A. Irizarry, and S. Dudoit, eds. (Springer), pp. 397–420.

Subramanian, A., Tamayo, P., Mootha, V.K., Mukherjee, S., Ebert, B.L., Gillette, M.A., Paulovich, A., Pomeroy, S.L., Golub, T.R., Lander, E.S., and Mesirov, J.P. (2005). Gene set enrichment analysis: a knowledge-based approach for interpreting genome-wide expression profiles. Proc. Natl. Acad. Sci. USA *102*, 15545–15550.

Sugimoto, K., Ogawa, A., Mizoguchi, E., Shimomura, Y., Andoh, A., Bhan, A.K., Blumberg, R.S., Xavier, R.J., and Mizoguchi, A. (2008). IL-22 ameliorates intestinal inflammation in a mouse model of ulcerative colitis. J. Clin. Invest. *118*, 534–544.

R Core Team. (2014). R: A language and environment for statistical computing (R Foundation for Statistical Computing).

Tilloy, F., Treiner, E., Park, S.H., Garcia, C., Lemonnier, F., de la Salle, H., Bendelac, A., Bonneville, M., and Lantz, O. (1999). An invariant T cell receptor alpha chain defines a novel TAP-independent major histocompatibility complex class Ib-restricted alpha/beta T cell subpopulation in mammals. J. Exp. Med. *189*, 1907–1921.

Treiner, E., Duban, L., Bahram, S., Radosavljevic, M., Wanner, V., Tilloy, F., Affaticati, P., Gilfillan, S., and Lantz, O. (2003). Selection of evolutionarily conserved mucosal-associated invariant T cells by MR1. Nature *422*, 164–169.

Ussher, J.E., Bilton, M., Attwod, E., Shadwell, J., Richardson, R., de Lara, C., Mettke, E., Kurioka, A., Hansen, T.H., Klenerman, P., and Willberg, C.B. (2014). CD161++ CD8+ T cells, including the MAIT cell subset, are specifically activated by IL-12+IL-18 in a TCR-independent manner. Eur. J. Immunol. *44*, 195–203.

Ussher, J.E., van Wilgenburg, B., Hannaway, R.F., Ruustal, K., Phalora, P., Kurioka, A., Hansen, T.H., Willberg, C.B., Phillips, R.E., and Klenerman, P. (2016). TLR signaling in human antigen-presenting cells regulates MR1-dependent activation of MAIT cells. Eur. J. Immunol. *46*, 1600–1614.

van Wilgenburg, B., Scherwitzl, I., Hutchinson, E.C., Leng, T., Kurioka, A., Kulicke, C., de Lara, C., Cole, S., Vasanawathana, S., Limpitikul, W., et al.; STOP-HCV consortium (2016). MAIT cells are activated during human viral infections. Nat. Commun. *7*, 11653.

van Wilgenburg, B., Loh, L., Chen, Z., Pediongco, T.J., Wang, H., Shi, M., Zhao, Z., Koutsakos, M., Nüssing, S., Sant, S., et al. (2018). MAIT cells contribute to protection against lethal influenza infection *in vivo*. Nat. Commun. *9*, 4706.

Vantourout, P., and Hayday, A. (2013). Six-of-the-best: unique contributions of γδ T cells to immunology. Nat. Rev. Immunol. *13*, 88–100.

Wang, H., D'Souza, C., Lim, X.Y., Kostenko, L., Pediongco, T.J., Eckle, S.B.G., Meehan, B.S., Shi, M., Wang, N., Li, S., et al. (2018). MAIT cells protect against pulmonary Legionella longbeachae infection. Nat. Commun. *9*, 3350.

Yamaguchi, H., Hirai, M., Kurosawa, Y., and Hashimoto, K. (1997). A highly conserved major histocompatibility complex class I-related gene in mammals. Biochem. Biophys. Res. Commun. *238*, 697–702.

Yu, G., and He, Q.Y. (2016). ReactomePA: an R/Bioconductor package for reactome pathway analysis and visualization. Mol. Biosyst. *12*, 477–479.

STAR★METHODS

KEY RESOURCES TABLE

REAGENT or RESOURCE	SOURCE	IDENTIFIER
Antibodies		
Anti Hu-CCR1 eFluor450; SF10B29; 1:100	Biolegend	362907; RRID: AB_2563918
Anti Hu-CCR2 FITC; K036C2; 1:100	Biolegend	357215; RRID: AB_2562945
Anti Hu-CCR4 PerCP Cy5.5; L291H4; 1:100	Biolegend	359405; RRID: AB_2562390
Anti Hu-CCR5 FITC; HEK/1/859; 1:100	Biolegend	313705; RRID: AB_345305
Anti Hu-CCR6 APC; G034E3; 1:100	Biolegend	353415; RRID: AB_10945155
Anti Hu-CCR7 APC-Cy7; G043H7; 1:100	Biolegend	353212; RRID: AB_10916390
Anti Hu-CD3 PEAF 594; UCHT1; 1:200	BD Bioscience	562280; RRID: AB_11153674
Anti Hu-CD3 FITC; HIT3a; 1:80	eBioscience	11-0039-42; RRID: AB_1724043
Anti Hu-CD3 PE; OKT3; 1:20	BD Bioscience	555333; RRID: AB_395740
Anti Hu-CD45RA APC; HI100; 1:50	eBioscience	48-0458-42; RRID: AB_1272059
Anti Hu-CD45RA FITC; HI100; 1:50	BD Bioscience	555488; RRID: AB_395879
Anti Hu-CD8a PerCP Cy5.5; SK1; 1:100	BD Bioscience	565310; RRID: AB_2687497
Anti Hu-CD8α APC; RPA-T8; 1:80	eBioscience	17-0088-42; RRID: AB_10669564
Anti Hu-CD8β APC; 2ST8.5H7; 1:80	BD Bioscience	641058; RRID: AB_1645723
Anti Hu-CXCR4 BV421; 12G5; 1:100	Biolegend	306517; RRID: AB_10901163
Anti Hu-CXCR6 BV421 K041E5; 1:100	Biolegend	356013; RRID: AB_25622514
Anti Hu-GM-CSF PE; BVD2-21C11; 1:40	Biolegend	502305; RRID: AB_2085533
Anti Hu-IFN-γ AF700; B27; 1:80	BD Bioscience	557995; RRID: AB_396977
Anti Hu-IFN-γ FITC; 25723.11; 1:50	BD Bioscience	340449; RRID: AB_400425
Anti Hu-IL-10 PE; JES3-9D7; 1:40	eBioscience	12-7108-82; RRID: AB_466179
Anti Hu-IL-17A PE-Cy7; eBio64DEC17; 1:50	eBioscience	25-7179-42; RRID: AB_11063994
Anti Hu-IL-17F AF488; Poly5166; 1:50	Biolegend	516603; RRID: AB_10730721
Anti Hu-LIF APC; REA350; 1:50	Miltenyi	130-105-513; RRID: AB_2652645
Anti Hu-TCR Vα7.2 APC; 3C10; 1:50	Biolegend	351708; RRID: AB_10933246
Anti Hu-TNF PE; mAb11; 1:50	eBioscience	12-7349-41; RRID: AB_10668834
Anti Ms-CD19 PerCP-Cy5.5; ID3; 1:200	BD Bioscience	551001; RRID: AB_394004
Anti Ms-CD4 APC-Cy7; GK1.5; 1:200	BD Bioscience	552051; RRID: AB_394331
Anti Ms-CD44 AF700; IM7; 1:50	BD Bioscience	560567; RRID: AB_1727480
Anti Ms-CD45.2 FITC; 104; 1:200	BD Bioscience	553772; RRID: AB_395041
Anti Ms-CD62L BV605; Mel-14; 1:100	BD Bioscience	563252; RRID: AB_2738098
Anti Ms-CD8α PE; 53-6.7; 1:800	BD Bioscience	553032; RRID: AB_394570
Anti Ms-CSF2 (GM-CSF) APC; MP1-22E9; 1:150	Biolegend	505414; RRID: AB_2721461
Anti Ms-GITRL (TNFSF18) PE; YGL386; 1:150	Biolegend	120305; RRID: AB_2287690
Anti Ms-IFN-γ PE; XMG1.2; 1:200	BD Bioscience	554412; RRID: AB_395376
Anti Ms-IFN-γ PE-Cy7; XMG1.2; 1:400	BD Bioscience	557649; RRID: AB_396766
Anti Ms-IL-10 PE; JES5-16E3; 1:300	eBioscience	12-7101-81; RRID: AB_466175
Anti Ms-IL-17A APC; 17B7; 1:25	eBioscience	17-7177-81; RRID: AB_763580
Anti Ms-IL-17F PE; 316016; 1:200	R&D	IC2057P; RRID: AB_2295980
Anti Ms-TCRβ APC; H57-597; 1:200	BD Bioscience	561080; RRID: AB_10584335
Anti Ms-TCRβ PE; H57-597; 1:200	BD Bioscience	561081; RRID: AB_10563767
Anti Ms-TCRβ PE CF594; H57-597; 1:200	BD Bioscience	562841; RRID: AB_2737831
Anti Ms-TNF PE; MP6-XT22; 1:300	BD Bioscience	554419; RRID: AB_395380
Anti Ms-TRANCE (TNFSF11)PE; IK22/5; 1:150	Biolegend	510005; RRID: AB_315553

(*Continued on next page*)

Continued

REAGENT or RESOURCE	SOURCE	IDENTIFIER
Bacterial and Virus Strains		
Legionella longbeachae	Clinical isolate NSW150 (Cazalet et al., 2010)	N/A
Biological Samples		
Healthy adult peripheral human blood mononuclear cells	Volunteer participants, Royal Melbourne Hospital	Ethics ID 2002.107
Chemicals, Peptides, and Recombinant Proteins		
Pam3CSK4	Invivogen	# tlrl-Pam3CSK4
Heat Killed *Listeria monocytogenes*	Invivogen	# tlrl-hklm
Poly I:C (high molecular weight)	Invivogen	# tlrl-Poly(I:C)(HMW)
Poly I:C (low molecular weight)	Invivogen	# tlrl-Poly(I:C)(LMW)
Lipopolysaccharide from *E.coli*	Enzo life sciences	ALX-581-012-L001
Flagellin from *S.typhimurium*	Invivogen	TLRL-EPSTELA
FSL-1 (Pam2CGDPKHPKSF)	Invivogen	# tlrl-FSL-1
Pam2Cys	Synthesized in house	N/A
Imiquimod	Novus biologicals	NBP2-26228
CpG ODN1826	Invivogen	# tlrl-ODN1826
Critical Commercial Assays		
Absolutely RNA Microprep Kit	Agilent	#400805
Fixation/Permeabilization Solution Kit	BD Biosciences	554714
QIAshredder	QIAGEN	79654
Bioanalyzer 2100 RNA pico kit	Agilent	5067-1512, 5067-1529, 5067-1511
Nextera XT library preparation	Illumina	FC-131-1002
AMPure XP	Beckman Coulter	A63880
SMART-Seq v4 Ultra Low Input RNA Kit for Sequencing	Clontech	634894
Deposited Data		
RNA Sequencing dataset 'Transcriptome of activated human and mouse MAIT cells' are deposited in Gene Expression Omnibus	This paper	GSE123805
Experimental Models: Organisms/Strains		
Mouse: C57BL/6	Biological Research Facility, Peter Doherty Institute, Melbourne	N/A
Oligonucleotides		
CpG 1668 T*C*C*A*T*G*A*C*G*T*T*C*C*T*G*A*T*G*C*T (*phosphorothioate linkage)	Geneworks	N/A
MR1 5′ 8763-8783 AGC TGA AGT CTT TCC AGA TCG	Geneworks	1204410
MR1 9188-9168 rev ACA GTC ACA CCT GAG TGG TTG	Geneworks	1204411
MR1 10451-10431 GAT TCT GTG AAC CCT TGC TTC	Geneworks	1204412
Software and Algorithms		
STAR Aligner Software	Dobin et al., 2013	https://github.com/alexdobin/STAR
Rsubread	Liao et al., 2013	https://bioconductor.org/packages/release/bioc/html/Rsubread.html
R package	R Core Team, 2014	https://www.r-project.org/
EdgeR R package	Robinson et al., 2010	https://bioconductor.riken.jp/packages/3.0/bioc/html/edgeR.html
ReactomePA	Yu and He, 2016; Fabregat et al., 2018	https://github.com/GuangchuangYu/ReactomePA
voom in *limma*	Smyth, 2016	https://www.bioconductor.org/packages//2.10/bioc/html/limma.html
GSEA version 3.0	Subramanian et al., 2005	http://software.broadinstitute.org/gsea/downloads.jsp

(*Continued on next page*)

Continued		
REAGENT or RESOURCE	SOURCE	IDENTIFIER
ComBat algorithm in *sva*	Leek et al., 2012	https://bioconductor.org/packages/release/bioc/html/sva.html
Prism GraphPad software (version 7.0)	GraphPad Software, San Diego, CA	https://www.graphpad.com/scientific-software/prism/
FlowJo10 software	TreeStar, Ashland, OR	https://www.flowjo.com/solutions/flowjo/downloads
Partek Flow	Partek Incorporated, St Louis, MO	http://www.partek.com/partek-flow/

LEAD CONTACT AND MATERIALS AVAILABILITY

Further information and requests for resources and reagents should be directed to and will be fulfilled by the Lead Contact, Timothy Hinks (timothy.hinks@ndm.ox.ac.uk).

EXPERIMENTAL MODEL AND SUBJECT DETAILS

Mice

C57BL/6JArc mice were bred and housed in the Biological Research Facility of the Peter Doherty Institute (Melbourne, Victoria, Australia). Male mice aged 6–12 weeks, housed under specific pathogen-free conditions were used in experiments, after approval by the University of Melbourne Animal Ethics Committee (1513661 and 1513712). Animals were group housed, and littermates within the same cage were randomly allocated to receive stimuli or control.

Human volunteers

Human peripheral blood mononuclear cells (PBMC) were obtained from healthy volunteer participants (University of Melbourne Human Research Ethics Committee 2002.107). Subjects were adults (Median age 40, range 24-48) of both sexes (3 female, 1 male) who provided written informed consent. Groups of samples of each cell type and experimental condition (Table S2) included 1 male and 2 female samples in all cases. As sample groups were contrasted with each other, rather than within a group, no effect of sex on results will be apparent in the datasets or analyses provided, and hence results are expected to be generalisable to both sexes.

Bacterial strains

Cultures of *Legionella longbeachae* NSW150 were grown at 37°C in buffered yeast extract (BYE) broth supplemented with 30-50 μg/ml streptomycin for 16 hours to log-phase (OD600 0.2-0.6) with shaking at 180 rpm. For the infecting inoculum, bacteria were re-inoculated in pre-warmed medium for a further 2–4 h culture (OD_{600} 0.2–0.6) with the estimation that 1 OD_{600} = 5x10^8/ml, sufficient bacteria were washed and diluted in phosphate buffered saline (PBS) with 2% BYE for i.n. delivery to mice. A sample of inoculum was plated onto (BYCE) with streptomycin for verification of bacterial concentration by counting colony-forming units.

METHOD DETAILS

Compounds, immunogens and tetramers

5-OP-RU was prepared as described previously (Mak et al., 2017). CpG1668 (Sequence: T*C*C*A*T*G*A*C*G*T*T*C*C*T*G*A*T*G*C*T (*phosphorothioate linkage) nonmethylated cytosine-guanosine oligonucleotides was purchased from Geneworks (Thebarton, Australia) and Pam2Cys was chemically synthesized and functionally verified in house. Other toll like receptor ligands are detailed in Table S1. Murine and human MR1 and β2-Microglobulin genes were expressed in *Escherichia coli* inclusion bodies, re-folded, and purified as described previously (Patel et al., 2013). MR1-5-OP-RU tetramers were generated as described previously (Corbett et al., 2014).

In vivo infection

Intranasal (i.n.) inoculation with a stimulatory MR1 ligand (76 pmol 5-OP-RU) or a non-activating MR1 ligand (76 pmol 6-FP) and TLR agonist (see Table S1) in a total 50 μL volume was performed on isofluorane-anaesthetised mice on day 0. Additional doses of the relevant MR1 ligand in 50 μL were administered on days 1, 2, and 4. Control mice received TLR agonists alone, in a total volume of 50 μl. For infection experiments mice were inoculated with 1-2 x10^4 CFU *Legionella longbeachae* (clinical isolate NSW150; Cazalet et al., 2010) in 50 μL PBS.

Mice were weighed daily and assessed visually for signs of disease, including inactivity, ruffled fur, labored breathing, and huddling behavior. Animals that had lost $\geq$ 15% of their original body weight and/or displayed evidence of pneumonia were euthanised.

Tissue processing

Mice were killed by CO_2 asphyxia, the heart perfused with 10 mL cold Roswell Park Memorial Media-1640 (RPMI, GIBCO) and lungs were taken. To prepare single-cell suspensions lungs were finely chopped with a scalpel blade and treated with 3 $mg.ml^{-1}$ collagenase III (Worthington, Lakewood, NJ), 5 μg/ml DNase, and 2% fetal calf serum in RPMI for 90 min at 37°C with gentle shaking, and, where relevant, brefeldin A (GolgiPlug, BD Biosciences, San Diego, CA). Lung cells were then filtered (70 μm) and washed with PBS/2% fetal calf serum. Red blood cells were lysed with hypotonic buffer TAC (Tris-based amino chloride) for 5 min at 37°C. Approximately $1.5x10^6$ cells were filtered (40 μm) and used for flow cytometric analysis. To obtain sufficient cells for sorting naive $CD8^+CD44^-CD62L^+$ cells from infection-naive mice lungs from 2-3 mice per sample were pooled and stained with 0.18 μL anti-CD8-PE, then magnetically enriched using anti-PE beads (Miltenyi) prior to sorting.

For analysis of systemic MAIT cell distribution lymphocytes were obtained from mesenteric lymph nodes by passing through a 70 μm strainer. Splenocytes were obtained by homogenizing splenic tissue through a 70 μm strainer then preforming red cell lysis prior to staining. Peripheral blood cells were obtained from the inferior vena cava into a heparinised syringe and underwent surface staining prior to red cell lysis with 1 mL of 10% red cell lysis buffer (BD Bioscience) for 5 minutes at room temperature before washing twice with FACS buffer. Hepatic lymphocytes were obtained by perfusing the liver with 8-10 mL PBS, passing through a 40 μm strainer, washing once with PBS the resuspending in 36% Percoll (Sigma) and centrifuging without braking at 800 *g* for 25 mins at RT over a 70% Percoll underlay. Cells from the interphase were washed with FACS buffer, red cells lysed with 1 mL 10% red cell lysis buffer, cells washed twice and stained for flow cytometry.

Determination of bacterial counts in infected lungs

Bacterial infection was determined for *L. longbeachae* by counting colony-forming units (CFU) obtained from plating homogenized lungs in duplicate from infected mice (x5 per group) on buffered charcoal yeast extract agar (BYCE) containing 30 μg/ml streptomycin and colonies counted after 4 days at 37°C under aerobic conditions.

Antibodies flow cytometry and cell sorting

Details of flow cytometry antibodies are shown in the Key Resources Table. To block non-specific staining, cells were incubated with MR1-6-FP tetramer and anti-Fc receptor (2.4G2) for 15 min at room temperature and then incubated at room temperature with Ab/tetramer cocktails in PBS/2% fetal calf serum. Dead cells were excluded using 4′,6-diamidino-2-phenylindole (DAPI) for live cell sorting added for 10 mins or by staining for 20 mins in PBS with fixable viability dyes Zombie Yellow (Biolegend, 1:100, 423104) or Live/Dead EF780 (BD Bioscience, 1:1000, 565388).

For live cell sorting on human peripheral blood mononuclear cells (PBMC) 50ml of heparinised blood were obtained freshly per volunteer, mixed with an equal volume of phosphate buffered saline (PBS) and layered over an equal volume of Ficoll-Paque (GE Healthcare, Chicago, IL) and centrifuged at 800 *g* for 20 mins at room temperature. Cells were washed twice with PBS, cells counted by trypan blue estimation, then half the cells were resuspended overnight in RPMI with 10% human serum for overnight rest and the other half resuspended in flow cytometry buffer comprising PBS with 2% fetal calf serum and 2 mM EDTA (FACS buffer) for immediate magnetic enrichment and sorting. These cells were stained with surface antibodies (CD3-PE-CF594, CD8-PerCPCy5.5, CD45RA-FITC, TCR Vα7.2-APC and FCγ block for 15 mins at RT, followed by staining with MR1-5-OP-RU tetramer-PE for 20 mins at RT. Tetramer positive cells were positively selected using anti-PE microbeads (10 μL per 10^{-7} cells, Miltenyi, Cologne, Germany) according to the manufacturer's instructions. Cells were sorted immediately into ice cold PBS with 10% FCS using an FACSAria III cell sorter (BD Bioscience) selecting live $CD3^+$TCR-Vα7.2^+MR1-5OP-RU-$tetramer^+$ MAIT cells from the positive fraction and live $CD3^+CD8^+CD45RA^+$MR1-$Tetramer^-$ cells from the negative fraction (Figure S7A). The following day the remaining cells were stimulated for 6 h with 10 nM 5-OP-RU then magnetically enriched using MR1-5-OP-RU-tetramer-PE and anti-PE microbeads, and live $CD3^+$TCR-Vα7.2^+MR1-5-OP-RU-$tetramer^+$ MAIT cells sorted in the same manner. Purity was checked and with an average of 98%. Immediately after sorting cells were centrifuged at 400 *g* for 5 mins then resuspended in 100 μL of RNA lysis buffer (Agilent Ltd, UK) with 0.7 μL β-mercaptoethanol and stored at −80°C.

For live cell sorting of murine T cells, CD8 cells were magnetically enriched using anti-CD8-PE and anti-PE microbeads and live $CD8^+CD44^-CD62L^+$ cells from uninfected mice, or live $CD3^+CD19^-CD45.2^+TCR\beta^+$MR1-5-OP-RU-$tetramer^+$ MAIT cells from previously infected mice were sorted as above (Figure S7B).

For intracellular staining, cells were fixed with 1% paraformaldehyde prior to analysis on LSRII or LSR Fortessa or Canto II (BD Biosciences) flow cytometers. For intracellular cytokine staining Golgi plug (BD Biosciences) was used during all processing steps. Cells stimulated with PMA (phorbol 12-myristate 13-acetate;)/ionomycin (20 ng ml^{-1}, 1 μg ml^{-1}, respectively) for 3 h at 37°C were included as positive controls. Surface staining was performed at 37°C, and cells were stained for intracellular cytokines using the BD Fixation/Permeabilization Kit (BD, Franklin Lakes, NJ) or transcription factors using the transcription buffer staining set (eBioscience) according to the manufacturers' instructions.

For validation of key targets identified by RNA sequencing flow cytometry was performed on cryopreserved human PBMC from additional healthy human donors. Samples were defrosted into pre-warmed RPMI with 10% human serum, stained with anti-TCR-Vα7.2-PE or anti-TCR-Vα7.2-PE and magnetically enriched using anti-PE or anti-APC microbeads. 200,000 positively-selected TCR-Vα7.2^+ cells or the negative fraction (for naive $CD8^+45RA^+$ cells) were co-cultured for 5 hours in the presence of brefeldin A with 100,000 class I reduced (C1R) antigen presenting cells (APCs) which had been previously pulsed for 2 hours with 10 nM 5-OP-RU, or

with naive C1R cells (unstimulated control), or with PMA / ionomycin (20 ng ml^{-1}, 1 μg ml^{-1}, respectively), or without any stimulation. Cells were then analyzed by surface and intracellular cytokine staining as above. For validation of murine targets cells were isolated from uninfected mice or mice which had undergone intranasal infection 7 days prior (acute) or 12 weeks prior (resolution) or reinfection 7 days prior, and cytometrically analyzed as described above.

RNA sequencing

Cells were lysed in Agilent lysis buffer (Agilent Ltd., UK) containing 100 mM β-mercaptoethanol and passed through a QIAshredder device (QIAGEN, Valencia, US), then RNA extracted using the Absolutely RNA Nanoprep Kit according to the manufacturer's instructions, including using of DNase I. RNA libraries were prepared at the Melbourne Translational Genomics Platform, Department of Pathology (The University of Melbourne). Briefly, RNA quality and quantity were assessed using the Bioanalyzer 2100 RNA pico kit (Agilent technologies). The input total RNA was normalized to 250 pg per sample and median RIN was 9.7 (range 5.7-10.0). RNA was reverse transcribed and cDNA amplified by *in vitro* transcription with the SMART-Seq v4 Ultra Low Input RNA Kit for Sequencing (Clontech). First strand cDNA synthesis and tailing by reverse transcription was performed using Clontech's proprietary SMART (Switching Mechanism at 5′ End of RNA Template) technology. Following first strand synthesis, cDNA was amplified 12 cycles by LD PCR using blocked PCR primers. Amplified cDNA was purified using AMPure XP prior to QC using the bioanalyser 2100 HS DNA kit (Agilent technologies). Library preparation of purified amplified cDNA was performed using Nextera XT library preparation (Illumina, AUS). Following QC, 150 pg of cDNA was tagmented (simultaneously fragmented with adaptors inserted) using Nextera transposons. Molecular barcodes were incorporated during 12 cycles of amplification followed by purification using AMPure XP. The libraries passed a quality checkpoint (Qubit and Bioanalyser HS DNA) prior to normalization and pooling before loading onto the HiSeq 2500 (Illumina, AUS) for paired end sequencing.

QUANTIFICATION AND STATISTICAL ANALYSIS

Quality control and analysis of RNA sequencing data

RNA-seq reads were aligned to reference genome sequences using STAR (Dobin et al., 2013) aligner software. Mapped reads were assigned to genomic features using *Rsubread* (Liao et al., 2013) R package (R Core Team, 2014).

Genes that were differentially expressed (> 2 fold, $p < 0.01$, FDR < 0.05) between conditions and their normalized expression values, were generated with *EdgeR* R package (Robinson et al., 2010), Partek® Flow®, an online analysis platform for Next Generation Sequencing data (http://www.partek.com/partek-flow/). Pathway enrichment analysis using the Reactome platform (Fabregat et al., 2018) was performed using *ReactomePA* (Yu and He, 2016) R package. Gene count data transform to log2-counts per million (logCPM) was performed using *voom* function in *limma* (Smyth, 2016) R package. Gene set enrichment analysis (GSEA) using *voom* transformed count data was performed using GSEA version 3.0 (Subramanian et al., 2005), comparing gene expression data as a whole with the reference gene list obtained from the publication by Linehan et al., (2018)

RNA-seq data (logCPM) and ImmGen microarray data were integrated using a common set of *Entrez* (Maglott et al., 2011) annotated genes; batch effect removal was performed using *ComBat* algorithm in *sva* (Leek et al., 2012) R package. Hierarchical clustering analysis of transcription profiles was conducted in R employing highly variable genes (IQR > 0.75; Gentleman et al., 2004) and Euclidian distance.

Standard statistical analysis

Statistical tests were performed using the Prism GraphPad software (version 7.0 La Jolla, CA). Normality of flow-cytometric data was confirmed for the majority of data columns using Shapiro-Wilk tests and comparisons between groups were performed using Student's t tests on untransformed (MAIT cell frequency) or log-transformed (absolute MAIT cell numbers) data as appropriate. Flow cytometric data analysis was performed with FlowJo10 software (Ashland, OR). Statistical analyses and samples sizes are specified in each figure legend. For standard statistical analyses a two-tailed P value (where relevant, adjusted for multiple comparisons) < 0.05 was considered statistically significant.

DATA AND CODE AVAILABILITY

The RNA Sequencing data have been deposited in the Gene Expression Omnibus (GEO) under accession number GSE123805.

Cell Reports, Volume *28*

Supplemental Information

Activation and *In Vivo* Evolution of the MAIT Cell Transcriptome in Mice and Humans Reveals Tissue Repair Functionality

Timothy S.C. Hinks, Emanuele Marchi, Maisha Jabeen, Moshe Olshansky, Ayako Kurioka, Troi J. Pediongco, Bronwyn S. Meehan, Lyudmila Kostenko, Stephen J. Turner, Alexandra J. Corbett, Zhenjun Chen, Paul Klenerman, and James McCluskey

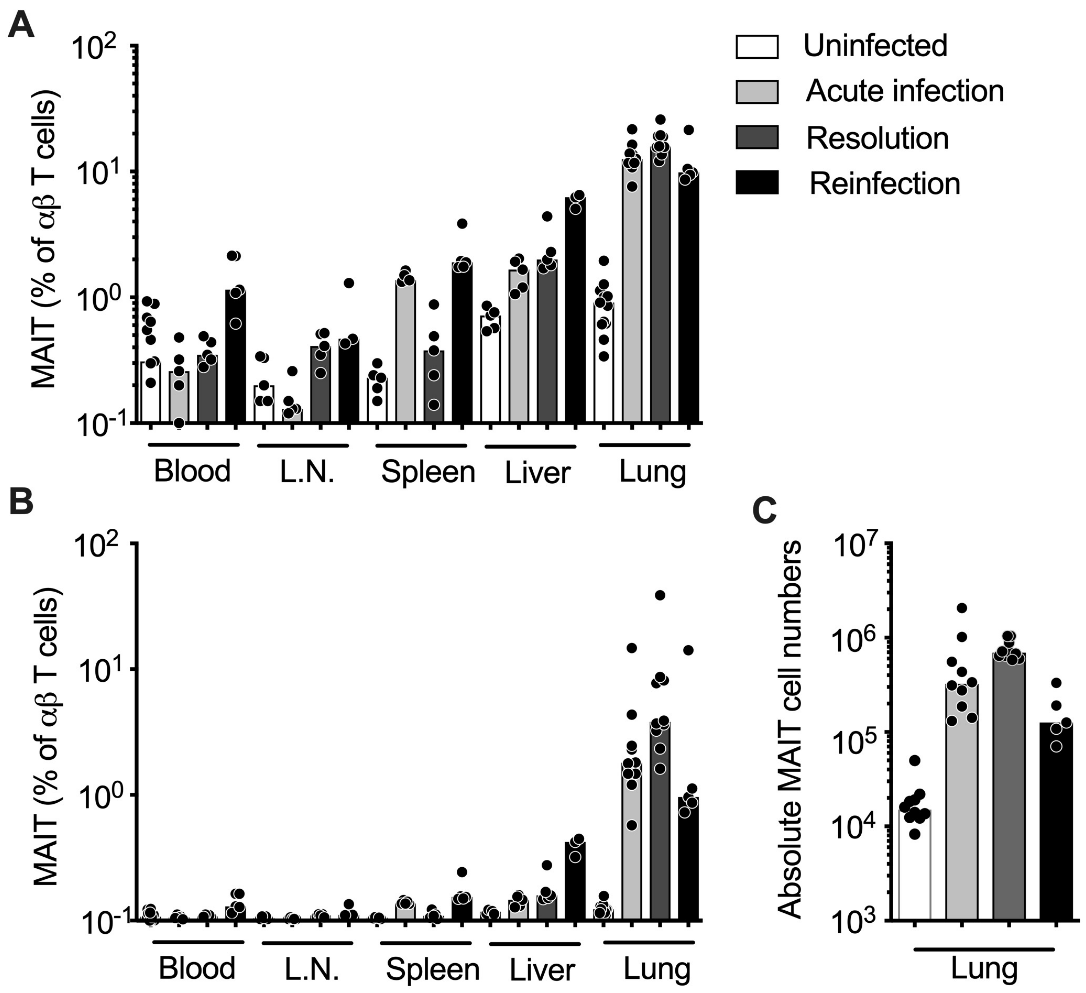

Supplementary figure S1. Tissue distribution of MAIT cells during infection *in vivo.* Related to Figure 1.

(**A, B**) Relative frequencies of MR1-5-OP-RU tetramer$^+$ MAIT cells as a proportion of total live TCRβ^+ T cells in the peripheral blood, mesenteric lymph node (L.N.), spleen, liver and lungs of C57BL/6 mice before, or after intranasal infection with 1x10^4 CFU *L. longbeachae*. Mice were sacrificed before ('uninfected'), or 7 days after infection ('acute'), or at least 12 weeks post infection ('resolution') or 7 days after a second intranasal infection with 2x10^4 CFU *L. longbeachae* in mice which had recovered from infection 12 weeks previously

(‘reinfection’). Graphs show combined data from experiments using 3-5 mice per group and performed one-three times. (**C**) Absolute numbers of pulmonary MAIT cells per mouse during the same experimental conditions, showing representative data from 5-10 mice per group performed twice. ANOVA $P<0.0001$, Tukey’s shows significant differences for all inter-group comparisons except acute v resolution.

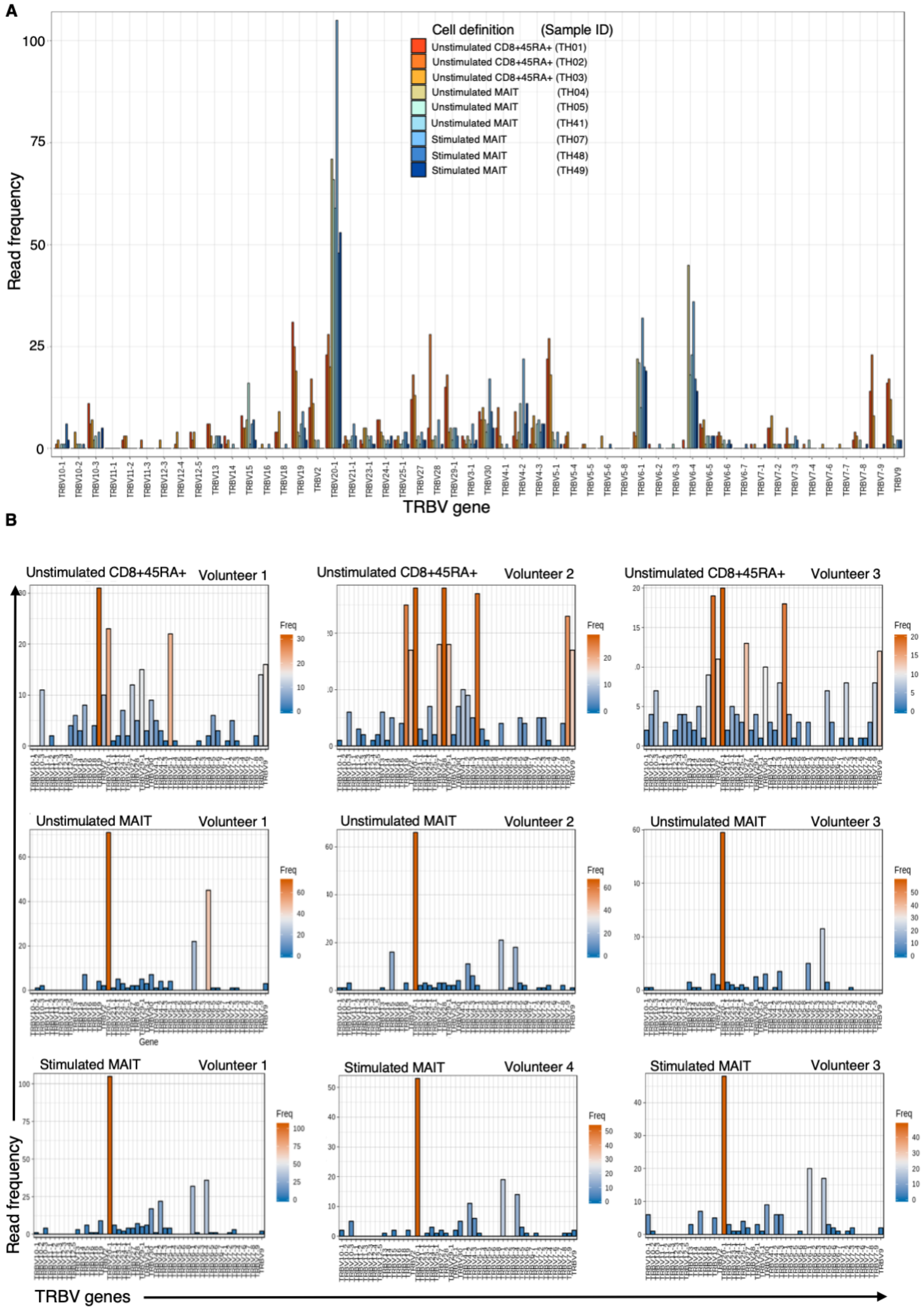

Supplementary figure S2. TCR repertoire analyses: human. Related to Table

2.

Analysis of TCR TRBV gene segment utilisation in human samples shown (**A**) aggregated and (**B**) for individual volunteers. RNA reads for TRBV genes from unstimulated human CD3+CD8+45RA+ T cells and MR1-5OP-RU-tetramer+ MAIT cells were compared for overall frequencies within the dataset. *y* axes show the absolute counts of gene segment usages in all clonetypes (unique CDR3 sequences identifying a unique cell clone) reconstructed. In MAIT cell populations increased read frequencies are observed for TRBV20-1, TRBV 6-1, and TRBV 6-4 in humans and for TRBV 19, TRBV 13-2 and TRBV 13-3 in mice, but with no specific repertoire changes observed between stages of infection. *n*=3 biological replicates / group performed once.

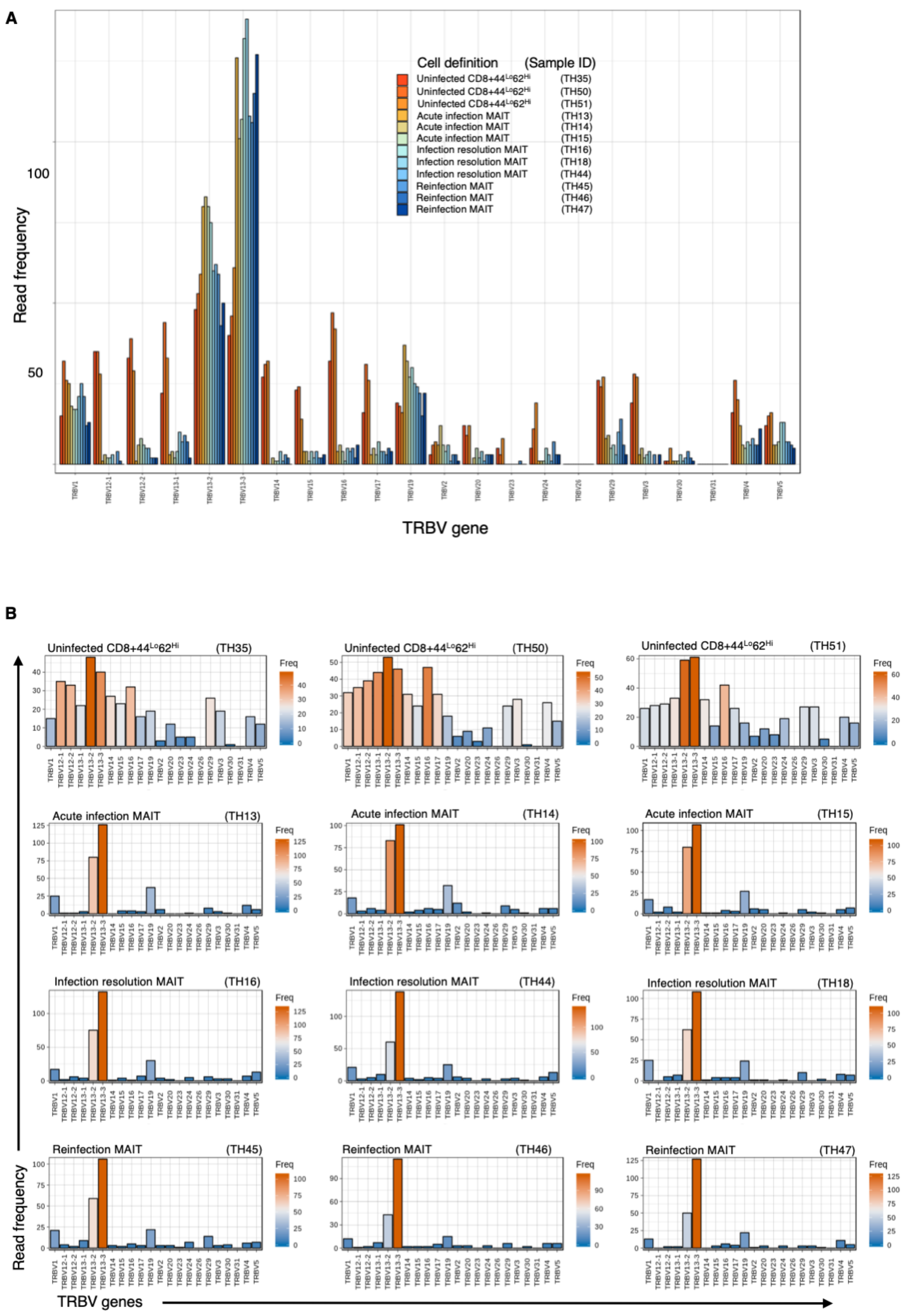

Supplementary figure S3. TCR repertoire: murine. Related to Table 2.

Analysis of TCR TRBV gene segment utilisation in murine samples shown (**A**) aggregated, where each naïve sample represents a pool from 3 animals, and (**B**) as individual replicates. RNA reads for TRBV genes from infection-naïve murine CD3+CD45.2+CD19-TCRβ+CD44-CD62L+ T cells and MR1-5OP-RU-tetramer+ MAIT cells were compared for overall frequencies within the dataset. *y* axes show the absolute counts of gene segment usages in all clonetypes (unique CDR3 sequences identifying a unique cell clone) reconstructed. In MAIT cell populations increased read frequencies are observed for TRBV 19, TRBV 13-2 and TRBV 13-3 in mice, but with no specific repertoire changes observed between stages of infection. *n*=3 biological replicates / group performed once.

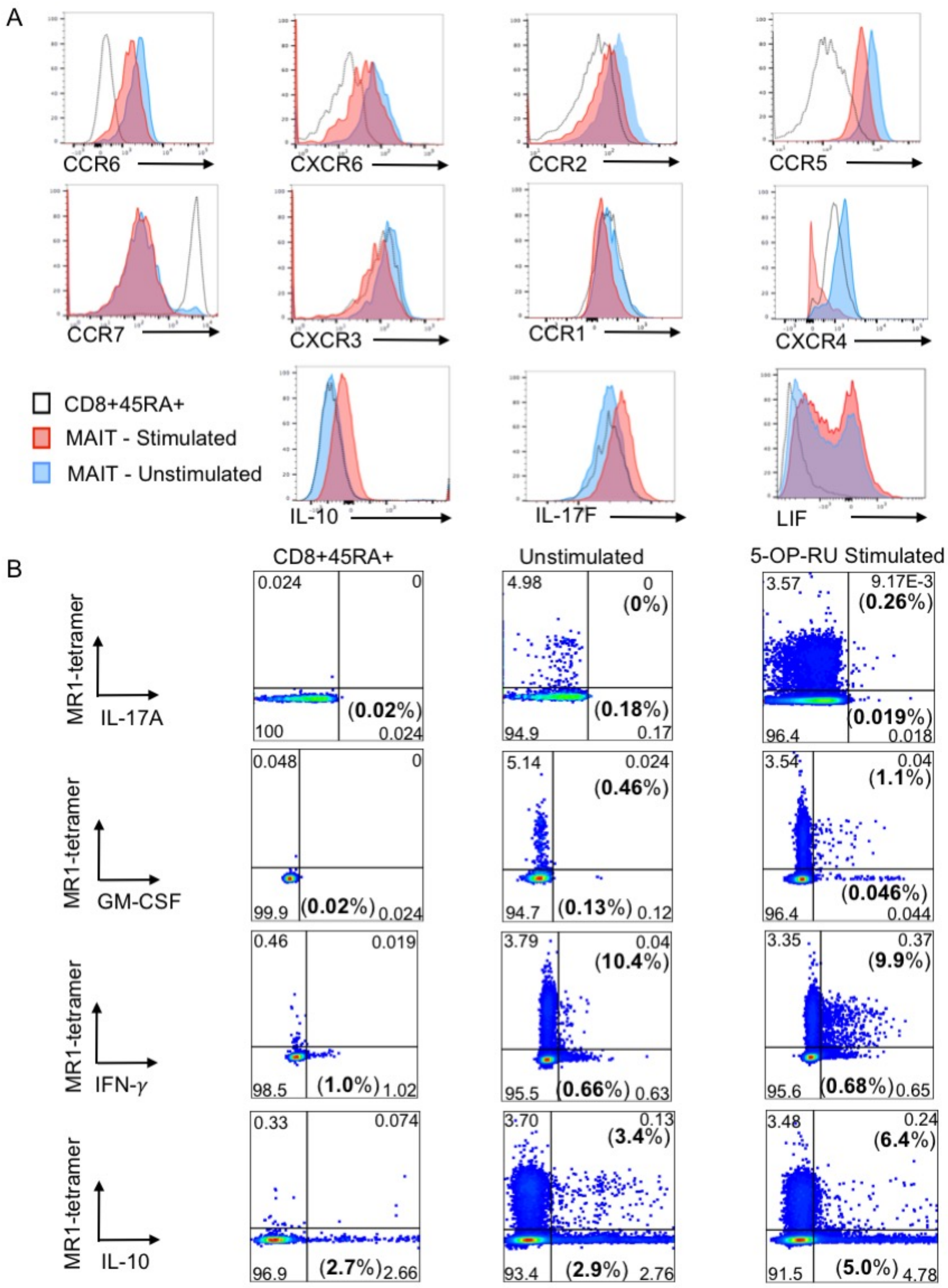

Supplementary figure S4. Cytometric validation of key differentially expressed genes (human). Related to Table 3.

(**A**) Representative flow-cytometry plots showing surface expression of the chemokine receptors CCR5, CCR7, CCR2, CCR1, CXCR6, CXCR4, CCR6, CXCR3, and intracellular

expression of the cytokines IL-10, IL-17F and LIF. Histograms compare staining of $CD8^+CD45RA^+$ cells (black, dotted) with unstimulated MAIT cells (blue) or MAIT cells after 6 h stimulation with 10 nM 5-OP-RU (red). (**B**) Representative flow-cytometry plots showing expression of the cytokines IL-17A, GM-CSF, IFN-γ and IL-10, by intracellular cytokine staining. Histograms compare staining of MR1-5-OP-RU $tetramer^+$ MAIT cells after 6 h stimulation with 10 nM 5-OP-RU in the presence of brefeldin A (right) with unstimulated cells (middle) and tetramer-negative, $CD8^+CD45RA^+$ naïve T cells (left). Figures in brackets represent percentage of MAIT cells (top / middle) or of $CD8^+CD45RA^+$ T cells (bottom) expressing the cytokine. Results are representative of three independent donors.

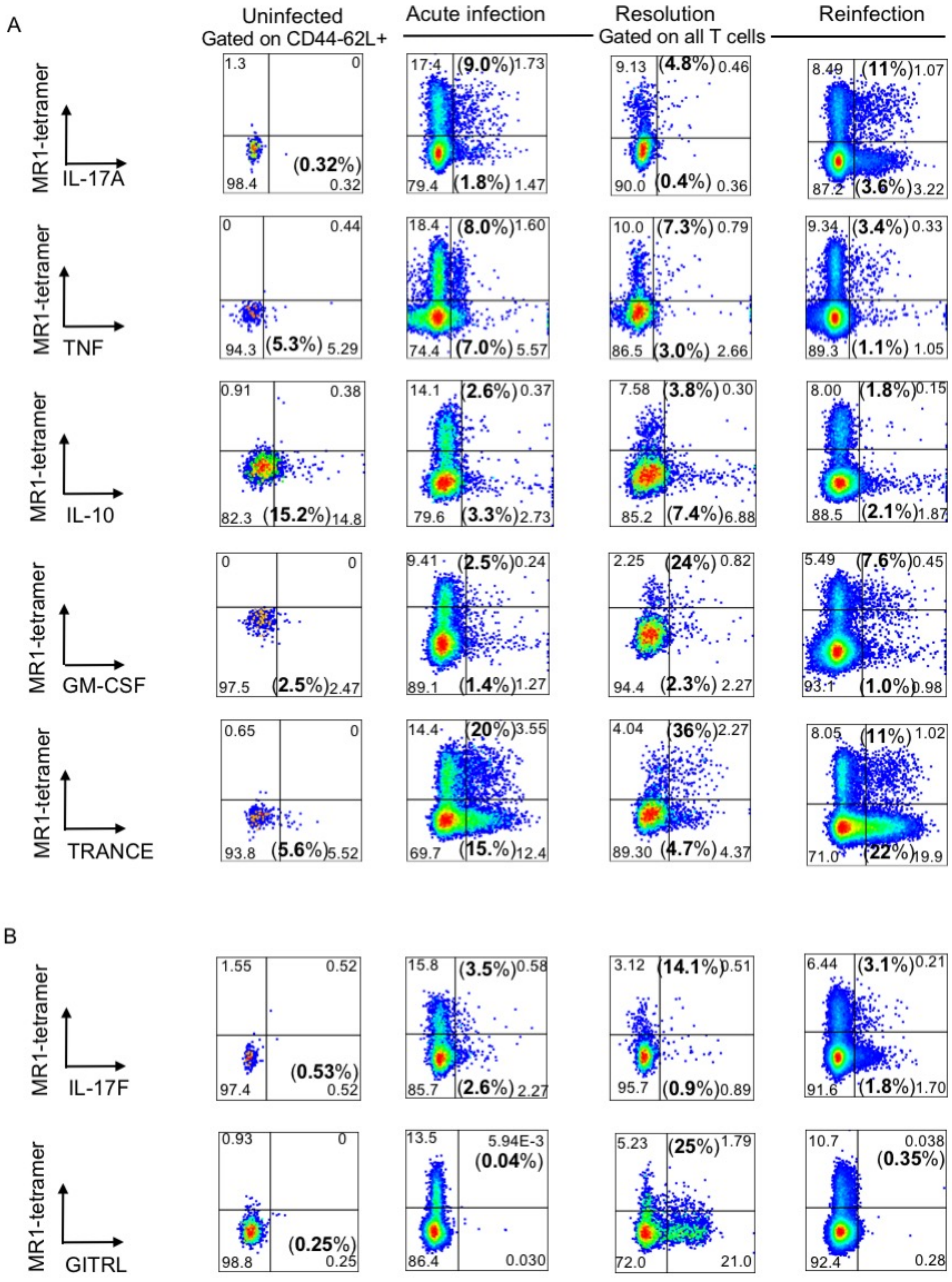

Supplementary figure S5. Cytometric validation of key differentially expressed genes (murine). Related to Table 2.

Representative flow-cytometry plots showing expression of the cytokines IL-17A, TNF, IL-10, TRANCE (TNFSF11), GM-CSF, IL-17F and GITRL (TNFSF18) by intracellular cytokine

staining on murine pulmonary T cells. Histograms compare staining of $CD44^-CD62L^+$ T cells from uninfected mice (left) with MR1-5-OP-RU tetramer$^+$ MAIT cells either 7 days ('acute', middle left) or 12 weeks ('resolution', middle right) after infection with 1 x10^4 CFU intranasal *L. longbeachae,* or 7 days after reinfection with 2 x10^4 CFU i.n. *L. longbeachae* in mice previously infected 12 weeks prior with 10^4 CFU i.n. *L. longbeachae* ('reinfection', right). Cells were incubated for 4 h in the presence of brefeldin A without (A, *ex vivo*) or with (B, stimulated) PMA and ionomycin. Figures in brackets represent percentage of $CD44^-CD62L^+$ T cells (left, lower quadrants) or MR1-tetramer$^+$ MAIT cells (middle and right, upper quadrants), or MR1-tetramer$^-$ conventional T cells (middle and right, lower quadrants) expressing the cytokine. Results are representative of three independent replicates performed on two separate days.

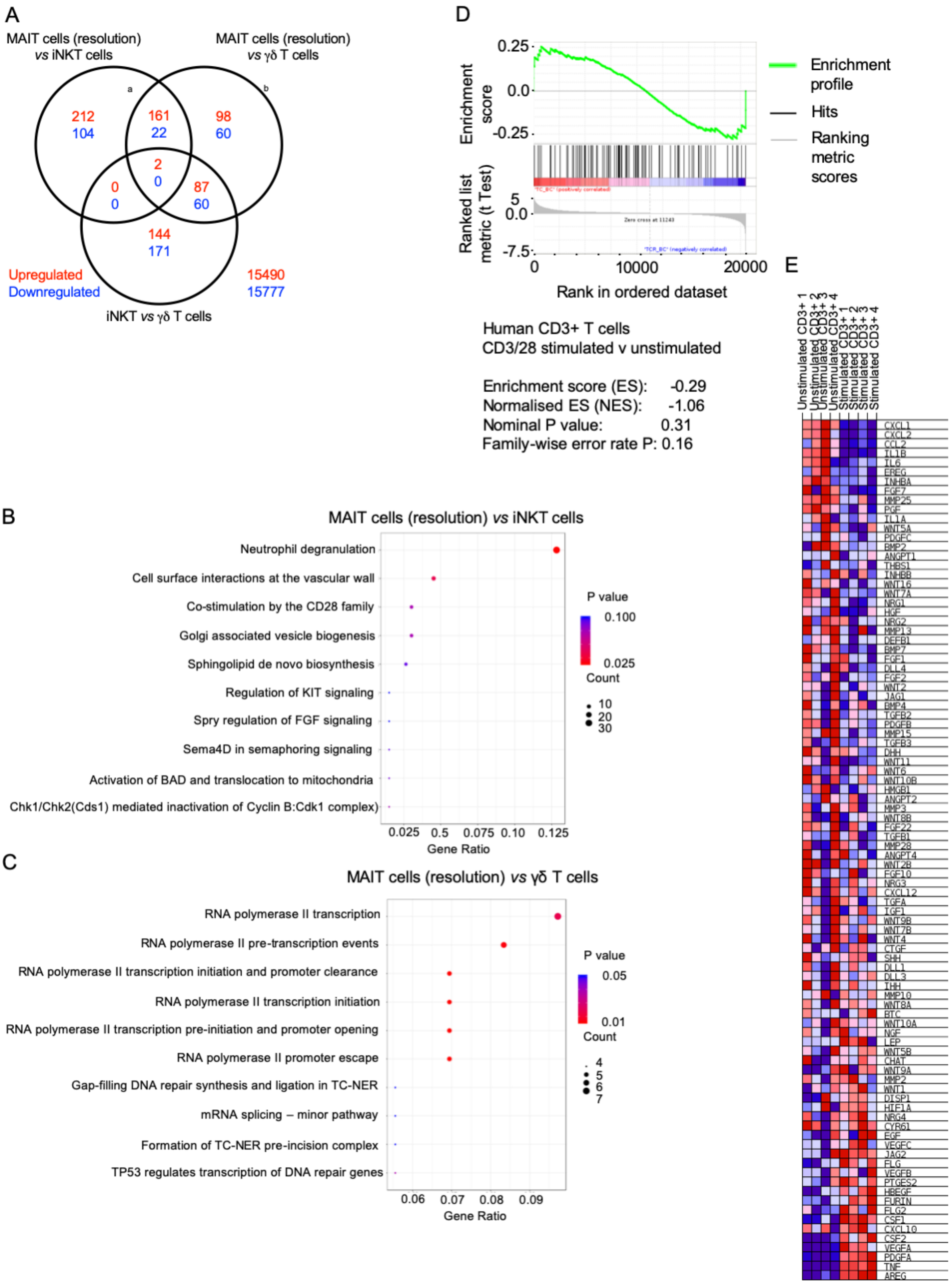

Supplementary figure S6. Additional analyses in murine MAIT, iNKT and γδ T cells, and human CD3+ cells. Related to Figure 2 and 4.

(A-C) Differential gene expression and Reactome pathway analysis comparing murine MAIT, iNKT and γδ T cells. (A) Venn diagram comparing numbers of differentially expressed genes between MAIT cells, iNKT cells and γδ T cells. Differential gene expression analysis was performed on transcriptomes of selected cell types shown in figure 3, comprising RNA seq data from this study and microarray data downloaded from the ImmGen database(Heng et al., 2008). MAIT cells comprised MR1-5-OP-RU tetramer+ MAIT cells at resolution of infection (12 weeks post infection). iNKT cells comprise all iNKT cell subsets shown in figure 3, excluding thymic precursor subsets: i.e. the ImmGen subsets: NKT.4-.Sp_1/2/3, NKT.4+/Sp1/2/3, NKT.4+.Lv_1/2/3/4, NKT.4-.Lv_1/2/3/4. Details of upregulated genes denoted (a) and (b) are provided in supplementary tables S15 and S16 respectively. (Red, upregulated; blue, downregulated). (**B**) Pathway analysis of the differential gene expression contrasts between murine lung MAIT cells at resolution of infection and non-thymic precursor iNKT cells shows that MAIT cells have upregulation of pathways associated with neutrophil degranulation and of cell surface interactions with vasculature. (**C**) Pathway analysis comparing murine lung MAIT cells at resolution of infection and γδ T cells. Upregulated MAIT cell pathways are those associated with RNA transcription. Plots show the extent to which named pathways from the curated Reactome database are upregulated. Colour intensity represents statistical significance of the upregulation, dot size represents the number of genes upregulated in the pathway, x axis represents the proportion of all differentially expressed genes included in the pathway ('gene ratio'). *n*=3 biological replicates / group performed once.

(**D-E**) Gene set enrichment analysis for tissue repair gene signature in human CD3+ T cells Gene set enrichment analysis (GSEA) was used to investigate potential enrichment of the same tissue repair signature presented in Figure 4(Linehan et al., 2018) in publicly available gene expression profiles from human CD3+ T cells (GEO Series accession no. GSE13887)(Fernandez et al., 2009). CD3+ T cells were negatively isolated with a Dynal kit

and, in the case of stimulated cells, Stimulated through CD3/CD28 for 24hr and gene expression analysed using Affymetrix Human Genome U133 Plus 2.0 Array. GSEA was performed on 4 replicates each of stimulated and unstimulated samples. (**A**) GSEA summary plots. The gene set is not significantly enriched: enrichment score (ES) = -0.28, normalised enrichment score (NES) = -1.06, family-wise error rate (FWER) p=0.16, nominal p value =0.3. (**B**) Heat map of expression of tissue repair genes (red, highest expression, blue, lowest). *n*=3 biological replicates / group performed once.

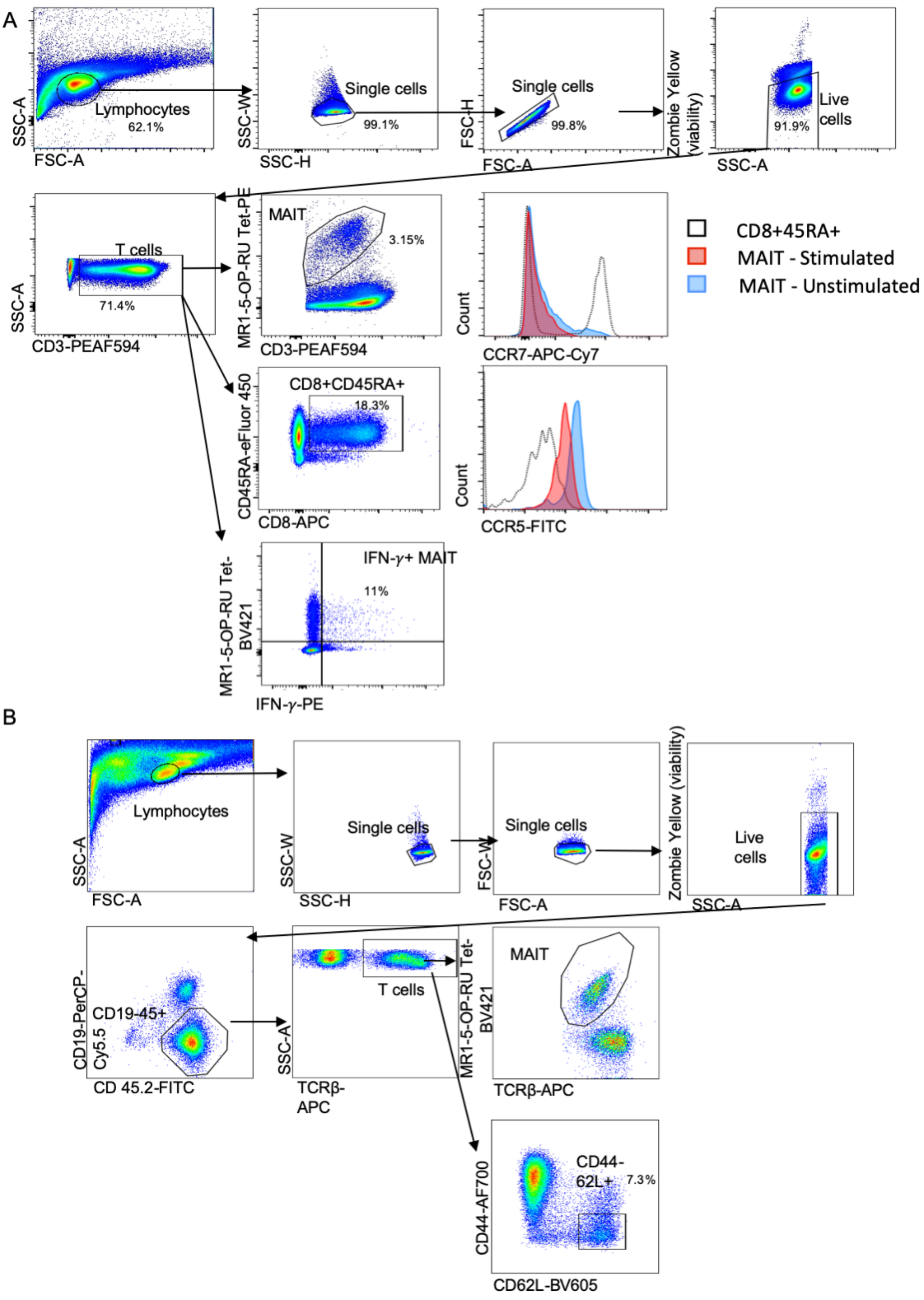

Supplementary figure S7. Cytometric gating strategies. Related to Table 1.

(**A**) Cytometric gating strategy (human). Human peripheral blood lymphocytes were identified, and doublets excluded, using forward and side scatter characteristics. Dead cells were excluded

using Zombie Yellow viability stain, then populations were gated on $CD3^+$ cells, then on either MR1-5-OP-RU tetramer conjugated to PE or BV421 for MAIT cells, or on CD8 and CD45RA to identify naïve $CD8^+CD45RA^+$ cells. Histograms compare staining of $CD8^+CD45RA^+$ cells (black) with unstimulated MAIT cells (blue) or MAIT cells after 6 h stimulation with 10 nM 5-OP-RU (red). For intracellular cytokines, where basal cytokine secretion was minimal, gates were set on the unstimulated MAIT cell sample.

(**B**) Cytometric gating strategy (murine). Related to Table 2. Pulmonary lymphocytes were identified, and doublets excluded, using forward and side scatter characteristics. Dead cells were excluded using Zombie Yellow viability stain, then populations were gated on $CD19^-$ $CD45.2^+$ cells, then TCRβ+ cells and finally MR1-5-OP-RU $tetramer^+$ MAIT cells, or $CD44^-$ $CD62L^+$ T cells.
